# A genetically informed Registered Report on adverse childhood experiences and mental health

**DOI:** 10.1038/s41562-022-01482-9

**Published:** 2022-12-08

**Authors:** Jessie R. Baldwin, Hannah M. Sallis, Tabea Schoeler, Mark J. Taylor, Alex S. F. Kwong, Jorim J. Tielbeek, Wikus Barkhuizen, Varun Warrier, Laura D. Howe, Andrea Danese, Eamon McCrory, Fruhling Rijsdijk, Henrik Larsson, Sebastian Lundström, Robert Karlsson, Paul Lichtenstein, Marcus Munafò, Jean-Baptiste Pingault

**Affiliations:** 1 Department of Clinical, Educational and Health Psychology, Division of Psychology and Language Sciences, University College London, London, UK; 2 Social, Genetic and Developmental Psychiatry Centre, Institute of Psychiatry, Psychology and Neuroscience, King’s College London, London, UK; 3 MRC Integrative Epidemiology Unit at the University of Bristol, Bristol Medical School, University of Bristol, Bristol, UK; 4 School of Psychological Science, University of Bristol, Bristol, UK; 5 NIHR Biomedical Research Centre, University Hospitals Bristol NHS Foundation Trust and University of Bristol, Bristol, UK; 6 Centre for Academic Mental Health, Population Health Sciences, Bristol Medical School, University of Bristol, Bristol, UK; 7 Department of Medical Epidemiology and Biostatistics, Karolinska Institutet, Nobels väg 12A, 171 77 Stockholm, Sweden; 8 Division of Psychiatry, Edinburgh Medical School, University of Edinburgh, Edinburgh, EH10 5HF, UK; 9 CNCR, Amsterdam Neuroscience Campus, VU University, Amsterdam, The Netherlands; 10 Department of Psychiatry, University of Cambridge, Cambridge, UK; 11 Department of Child & Adolescent Psychiatry, Institute of Psychiatry, Psychology & Neuroscience, King’s College London, London SE5 8AF, United Kingdom; 12 National and Specialist CAMHS Trauma, Anxiety, and Depression Clinic, South London and Maudsley NHS Foundation Trust, London, UK; 13 Anna Freud National Centre for Children and Families, London, UK; 14 Psychology Department, Faculty of Social Sciences, Anton de Kom University, Paramaribo, Suriname; 15 School of Medical Sciences, Örebro University, Örebro, Sweden; 16 Gillberg Neuropsychiatry Centre, Institute of Neuroscience and Physiology, University of Gothenburg, Gothenburg, Sweden; 17 Centre for Ethics, Law and Mental Health (CELAM), Institute of Neuroscience and Physiology, University of Gothenburg, Gothenburg, Sweden

## Abstract

Children who experience adversities have an elevated risk of mental health problems. However, the extent to which adverse childhood experiences (ACEs) cause mental health problems remains unclear, as previous associations may partly reflect genetic confounding. In this Registered Report, we used DNA from 11,407 children from the UK and USA to investigate gene-environment correlations and genetic confounding of the associations between ACEs and mental health. Regarding gene-environment correlations, children with higher polygenic scores for mental health problems had a small increase in odds for ACEs. Regarding genetic confounding, elevated risk of mental health problems in children exposed to ACEs was at least partially due to pre-existing genetic risk. However, some ACEs (e.g., childhood maltreatment, parental mental illness) remained associated with mental health problems independent of genetic confounding. These findings suggest that interventions addressing heritable psychiatric vulnerabilities in children exposed to ACEs may help to reduce their risk of mental health problems.

## Introduction

Adverse childhood experiences (ACEs) are well established risk factors for mental health problems. For example, a wealth of research has shown that children exposed to abuse, neglect, and dysfunctional home environments (such as domestic violence, parental separation, parental mental illness, criminal behaviour, or parental substance abuse) have a higher risk of developing internalising disorders such as depression and anxiety^1–4^, and externalising disorders such as conduct disorder and attention-deficit hyperactivity disorder (ADHD)^5–7^. However, as highlighted recently by policy makers^8^, charities^9^, and scientists^10,11^, the extent to which ACEs cause mental health problems is not known. This is because ACEs are not randomly distributed in the population, and children exposed to ACEs are likely to have other risk factors for mental health problems. In addition to wider environmental risks, one key potential vulnerability is genetic liability to mental health problems^12^.

There are at least two reasons why children exposed to ACEs might have an elevated genetic liability to mental health problems. First, parents with mental health problems may pass on genetic variants conferring psychopathology risk to their child and provide them with an adverse rearing environment. This represents a ‘passive gene-environment correlation’^13,14^, and is plausible as parental mental illness is considered to be an ACE, and other ACEs often occur in families where parents have mental health difficulties^15^. Second, a child with early phenotypic expressions of genetic liability to mental health problems might be more likely to elicit harsh parenting or stress responses in their parents (e.g., depressive symptoms). This represents an ‘evocative gene-environment correlation’^13,14^ and has been evidenced in adoption studies, where children at genetic risk of externalising problems were more likely to experience negative parenting from adoptive parents^16,17^. Importantly, if children with increased genetic liability to mental health problems have an elevated risk of experiencing ACEs, the association between ACEs and mental health problems may partly reflect genetic confounding.

It is important to investigate the extent to which genetic influences contribute to associations between ACEs and mental health to provide insights into causality and interventions. For example, if the associations are partly confounded by genetic influences, then the causal contribution of ACEs to mental health is likely to be lower than estimated in non-genetically informative studies. If this is the case, then even if we succeeded in implementing effective primary prevention of ACEs, this would only partly reduce children’s risk of mental health problems. In addition, secondary preventative strategies that support exposed children and address heritable vulnerabilities to psychopathology would be needed to reduce their risk of developing mental health problems. For example, this could include skills building components to manage negative emotions and behaviours as part of trauma-focused cognitive behavioural therapy^9^. Of course, there is a moral imperative to reduce the likelihood that children will experience ACEs, regardless of the degree to which they impact mental health. However, this research can improve our mechanistic understanding of the relationship between ACEs and mental health in ways that can help optimise approaches to prevention and intervention.

To examine the extent to which genetic influences contribute to associations between ACEs and mental health, particular genetically informed methods are needed. Twin methods (which have traditionally been used to test for genetic confounding)^18,19^ can be limited because many ACEs affect all children in a family, and thus, twins typically do not differ for the exposure. In addition, the adoption design (which can rule out genetic confounding due to passive gene-environment correlation) has limited utility because ACEs are rare in adoptive families^20^. Fortunately, recent advances in genome-wide association studies have allowed us to assess genetic influences in samples of unrelated individuals though polygenic scores. Polygenic scores capture common genetic influences by summing the effects of many genetic variants (known as single nucleotide polymorphisms; SNPs) on a trait into a single individual-level score. Through using polygenic scores, we can test whether (1) children with increased genetic liability to mental health problems are more likely to be exposed to ACEs (i.e., gene-environment correlation), and (2) such genetic influences contribute to the associations between ACEs and mental health (i.e., genetic confounding).

To examine gene-environment correlation, we can test whether a child’s polygenic score for a mental health problem (e.g., depression) predicts their exposure to ACEs. Three prospective studies employing this method have suggested that children with genetic liability to mental health problems may be more likely to experience ACEs. First, Sallis and colleagues^21^ found that children with higher polygenic scores for schizophrenia, ADHD, bipolar disorder, depression, and neuroticism had greater risk of exposure to broadly defined childhood trauma (including maltreatment, bullying, and domestic violence), with each standard deviation increase in the polygenic score predicting childhood trauma with odds ratios ranging between 1.07 (bipolar disorder) to 1.16 (depression). Second, Zwicker and colleages^22^ found that young people exposed to higher levels of broadly defined childhood adversity (including maltreatment, bullying and domestic violence) had higher polygenic scores for ADHD (standardised β=0.24), but not schizophrenia. Third, Schoeler and colleagues^23^ found that polygenic scores for depression, ADHD and risk taking (as well as body mass index and intelligence) independently predicted exposure to bullying victimisation in a multi-polygenic score model (with standardised βs ranging from 0.04 [risk taking] to 0.07 [depression]). These findings are also consistent with evidence from retrospective studies showing that adults reporting childhood maltreatment had higher polygenic scores for depression, schizophrenia, and bipolar disorder (with odds ratios ranging from 1.03 [bipolar disorder] to 1.20 [depression])^24,25^ as well as autism (standardised β=0.03)^26^. However, no study has systematically tested whether polygenic scores for a range of mental health problems predict a range of different ACEs, including indicators of household dysfunction (e.g., domestic violence, parental separation, parental mental illness, criminal behaviour, or parental substance abuse) as well as maltreatment. As such, it is not known whether some ACEs are more strongly linked to genetic risk of mental health problems than others, and whether certain genetic liabilities are particularly important in risk of exposure to ACEs.

To examine genetic confounding, we can test the extent to which the associations between ACEs and mental health are reduced when accounting for children’s polygenic scores for mental health problems^27^. To date, no study has examined whether this is the case for the associations between ACEs and mental health. However, studies have examined whether this is the case for related environmental experiences, such as adoption and parenting. With regard to adoption, Lehto and colleagues^28^ found that the associations between adoption and mental health-related outcomes in adulthood (depressive symptoms, bipolar disorder, neuroticism, and life satisfaction) were attenuated by between 3% (for bipolar disorder) to 18% (for life satisfaction) when controlling for the respective polygenic scores. With regard to parenting, Wertz and colleagues^29^ found that the associations between cognitive stimulation, warm, sensitive parenting, household chaos, and a safe, tidy home environment with child educational attainment were reduced by approximately 8% when controlling for the child’s polygenic score for education. Furthermore, Krapohl and colleagues^30^ found that the associations between parental slapping/smacking with ADHD and conduct problems were attenuated by 6% and 7%, respectively, when controlling for the child’s polygenic score for educational attainment.

Controlling for polygenic scores for mental health problems in this manner can indicate whether there is likely to be a genetic contribution to the association between ACEs and mental health. However, one limitation of this methodological approach is that polygenic scores only capture a small proportion of heritability, and thus do not fully account for genetic confounding. This can be addressed by a newly developed genetic sensitivity analysis^27^ which estimates shared genetic effects under scenarios in which the polygenic score captures additional genetic variance in the outcome (i.e., SNP- and/or twin-based heritability; see ‘Analysis plan’ section in the Methods for a detailed description of this method). A recent application of this genetic sensitivity analysis found that the associations between maternal education with offspring ADHD, educational achievement, and body mass index (BMI) were moderately explained by shared genetic effects^27^, consistent with findings from Children of Twins studies and adoption designs^31^. For example a latent polygenic score that captured SNP-based heritability in educational achievement (i.e., 31%^32^) explained 50% of the association between maternal education and child educational achievement^27^. However, this approach has never been applied to assess the extent to which genetic influences contribute to the associations between ACEs and mental health.

In this study, we systematically investigated the role of genetic liability in the associations between ACEs and mental health problems. To do so, we used data from more than 11,000 genotyped children from two cohorts in the United Kingdom (the Avon Longitudinal Study of Parents and Children [ALSPAC]) and the United States (the Adolescent Brain and Cognitive Development [ABCD] Study), with prospective measures of ACEs and mental health. (Note that the ABCD Study was not originally included in the Stage 1 pre-registration, but we used it because the original dataset, the Child and Adolescent Twin Study in Sweden [CATSS], was not accessible after Stage 1 acceptance [detailed in “Methods”]). We addressed the following aims and hypotheses (summarised in Table 1).

To examine gene-environment correlations, we investigated whether children with genetic liability to mental health problems are more likely to be exposed to ACEs (Aim 1). We addressed this by testing three hypotheses. First, we tested whether polygenic scores for mental health problems (e.g., depression, ADHD, schizophrenia, and others) are associated with exposure to ACEs. We hypothesised that polygenic scores for mental health problems would be associated with an increased risk of exposure to ACEs (Hypothesis 1a). Second, we tested whether polygenic scores for certain mental health problems are more strongly associated with ACEs than other polygenic scores. We hypothesised that there would not be evidence for differential associations between polygenic scores for different mental health problems with ACEs (Hypothesis 1b), given that previous research has identified similar size bivariate associations between a range of polygenic scores and ACEs^21^. Third, we tested whether certain ACEs are linked to greater polygenic risk for mental health problems than other ACEs. We hypothesised that parental mental illness, parental substance abuse, and parental criminality would be associated with higher polygenic risk for mental health problems relative to maltreatment, domestic violence, and parental separation (Hypothesis 1c), because the former exposures are most likely to be linked to intergenerational transmission of genetic risk for psychopathology.

To examine genetic confounding, we investigated the extent to which genetic liability to mental health problems contributes to the associations between ACEs and mental health (Aim 2). We addressed this by testing two hypotheses. First, we examined the proportions of the associations between ACEs and internalising and externalising problems that are explained by observed polygenic scores for mental health problems. We hypothesised that observed polygenic scores would explain a small proportion (between 5% to 20%) of the associations between ACEs and internalising and externalising problems (Hypothesis 2a) , given that a similar proportion of covariation between other early environments (adoption and parental discipline) and psychopathology were captured by polygenic scores^29,30^. Second, we estimated the proportions of the associations between ACEs and internalising and externalising problems that would be explained by latent polygenic scores which capture additional heritability in mental health problems. We hypothesised that polygenic scores that capture SNP heritability in internalising and externalising problems would explain a moderate proportion (between 20% to 40%) of the associations between ACEs and these outcomes (Hypothesis 2b). This is based on evidence showing that accounting for SNP heritability in an outcome can increase the covariance captured in an association by more than double, relative to a standard polygenic score^27^.[Table T1]

## Results

### Sample description

After imputation, the samples included 6,411 participants from ALSPAC and 4,996 participants from the ABCD Study. (Note that the ABCD Study was not originally included in the Stage 1 pre-registration, but we used it because the original dataset, the Child and Adolescent Twin Study in Sweden [CATSS], was not accessible after Stage 1 acceptance. Further information on the change in sample from CATSS to ABCD is reported in “Methods – Change in replication cohort”). Descriptive statistics are shown in Supplementary Table 1. Below we report results for the imputed samples, before testing whether findings replicate in the complete case samples (n=4,106 in ALSPAC and n=4,662 in ABCD).

### 1a) Do children with genetic liability to mental health problems have an increased risk of ACEs?

#### ALSPAC

**Figure 1:**
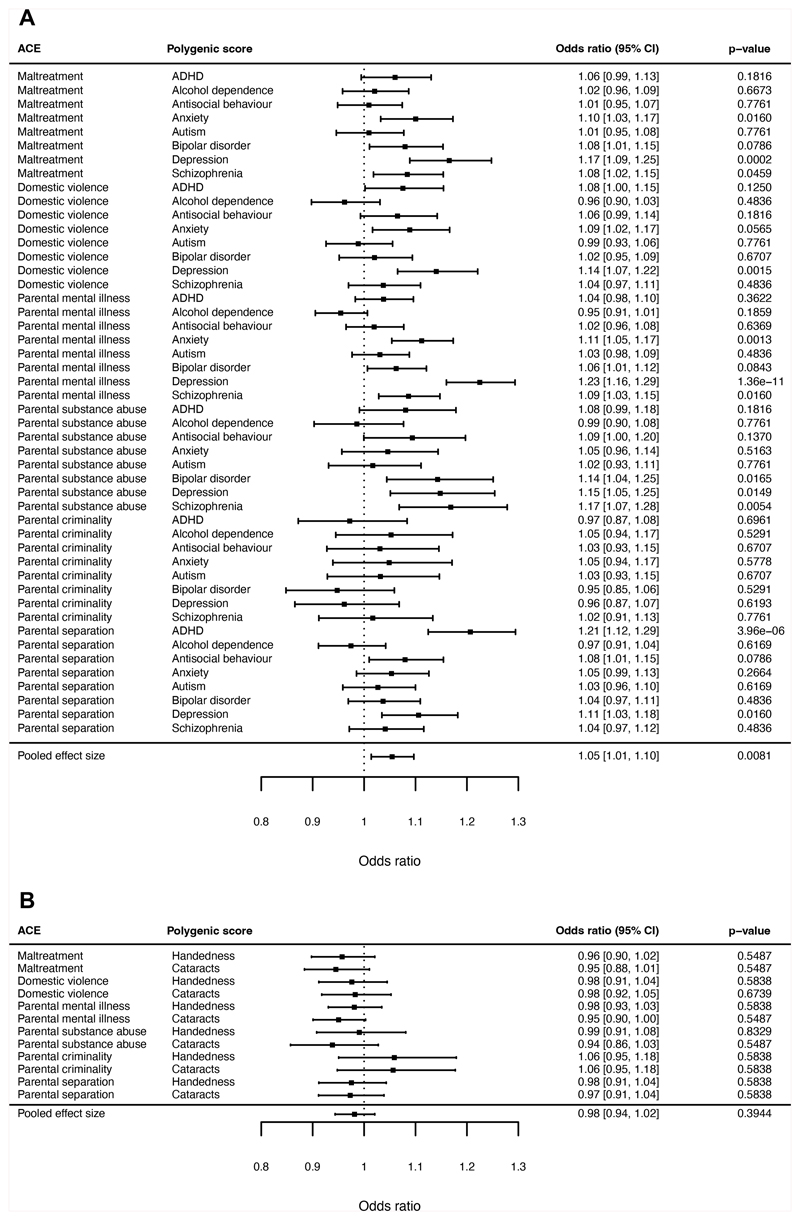
Associations between polygenic scores and ACEs in ALSPAC. Note. Data are presented as odds ratios +/- 95% CIs, obtained from logistic regression models. Panel A shows associations between polygenic scores for mental health problems and ACEs, Panel B shows associations between negative control polygenic scores and ACEs. P-values for individual associations between polygenic scores and ACEs are from two-sided tests and are false discovery rate (FDR) corrected. The sample size for ALSPAC analyses was n=6,411.

We first tested the associations between polygenic scores for mental health problems (depression, anxiety, bipolar disorder, autism, ADHD, antisocial behaviour, alcohol use disorder, and schizophrenia) and individual ACEs (maltreatment, domestic violence, parental mental illness, parental substance abuse, parental separation, and parental criminality). To obtain a single effect size reflecting the average association between polygenic scores for mental health problems and ACEs, we pooled the results across all individual associations. On average, we found that children from ALSPAC with higher polygenic scores for mental health problems had a small increase in odds of ACEs (pooled OR=1.05, 95% CI=1.01-1.10, p=0.0081; Figure 1A). To examine whether this effect size was trivially small, we performed equivalence tests, which assess whether the 90% confidence intervals for the effect size lie entirely within pre-specified equivalence bounds of OR=0.94-1.06 (indexing the smallest effect size of interest; see Methods, “Analysis plan”). The 90% CIs for the pooled association between polygenic scores for mental health problems and ACEs (1.02-1.09) did not fall completely within the equivalence bounds, suggesting the association was of meaningful magnitude. In contrast, negative control polygenic scores for handedness and cataracts were not associated with ACEs (pooled OR=0.98, 95% CI=0.94-1.02, p=0.39; Figure 1B).

#### ABCD

**Figure 2:**
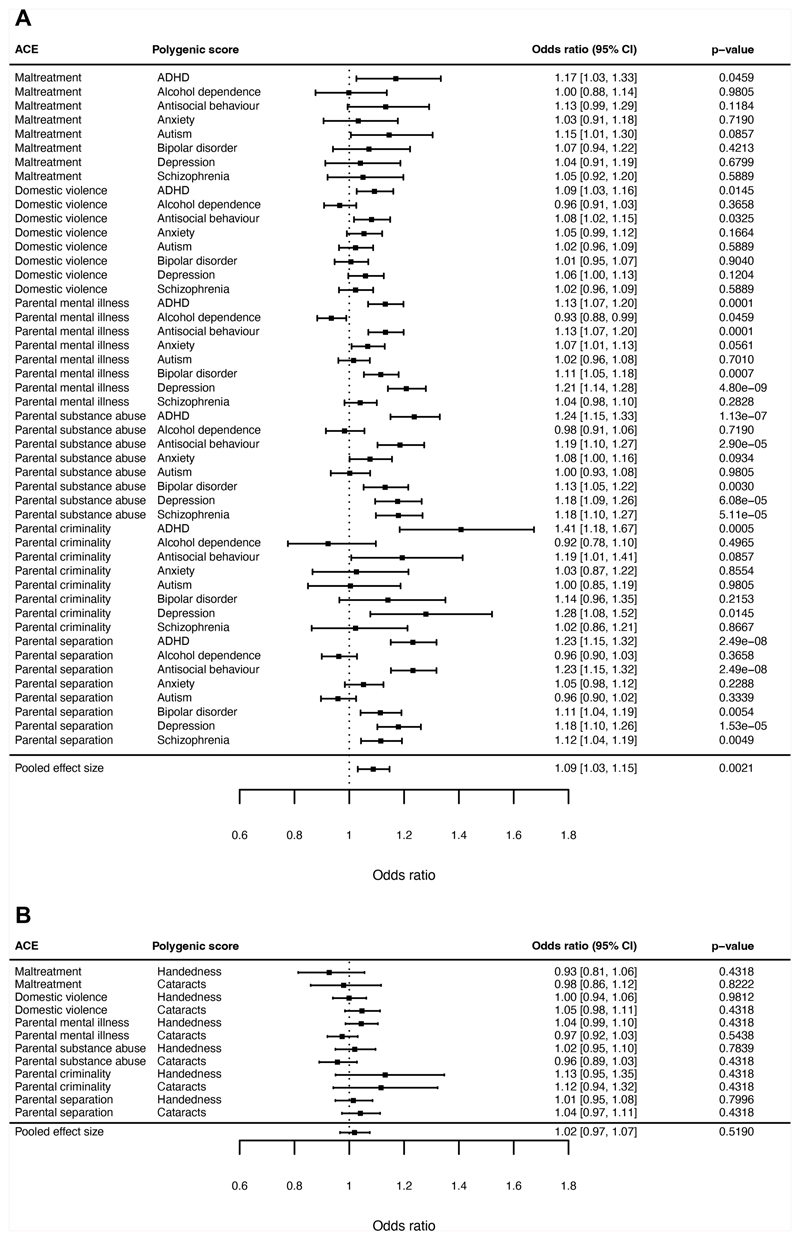
Associations between polygenic scores and ACEs in ABCD. Note. Data are presented as odds ratios +/- 95% CIs, obtained from logistic regression models. Panel A shows associations between polygenic scores for mental health problems and ACEs, Panel B shows associations between negative control polygenic scores and ACEs. P-values for individual associations between polygenic scores and ACEs are from two-sided tests and are FDR corrected. The sample size for ABCD analyses was n=4,996.

Similar to the ALSPAC Study, children in the ABCD cohort with greater polygenic scores for mental health problems had a small increase in odds of ACEs (pooled OR=1.09, 95% CI=1.03-1.15, p=0.0021, Figure 2A), and the 90% CIs (1.04-1.14) did not fall completely within the equivalence bounds (0.94-1.06). Conversely, negative control polygenic scores were not associated with ACEs (pooled OR=1.02, 95% CI=0.97-1.07, p=0.52; Figure 2B).

Taken together, findings from both cohorts pooled across ACEs support the hypothesis that polygenic scores for mental health problems are associated with an increased risk of exposure to ACEs.

### 1b) Are polygenic scores for certain mental health problems more strongly associated with ACEs than other polygenic scores?

#### ALSPAC

**Figure 3:**
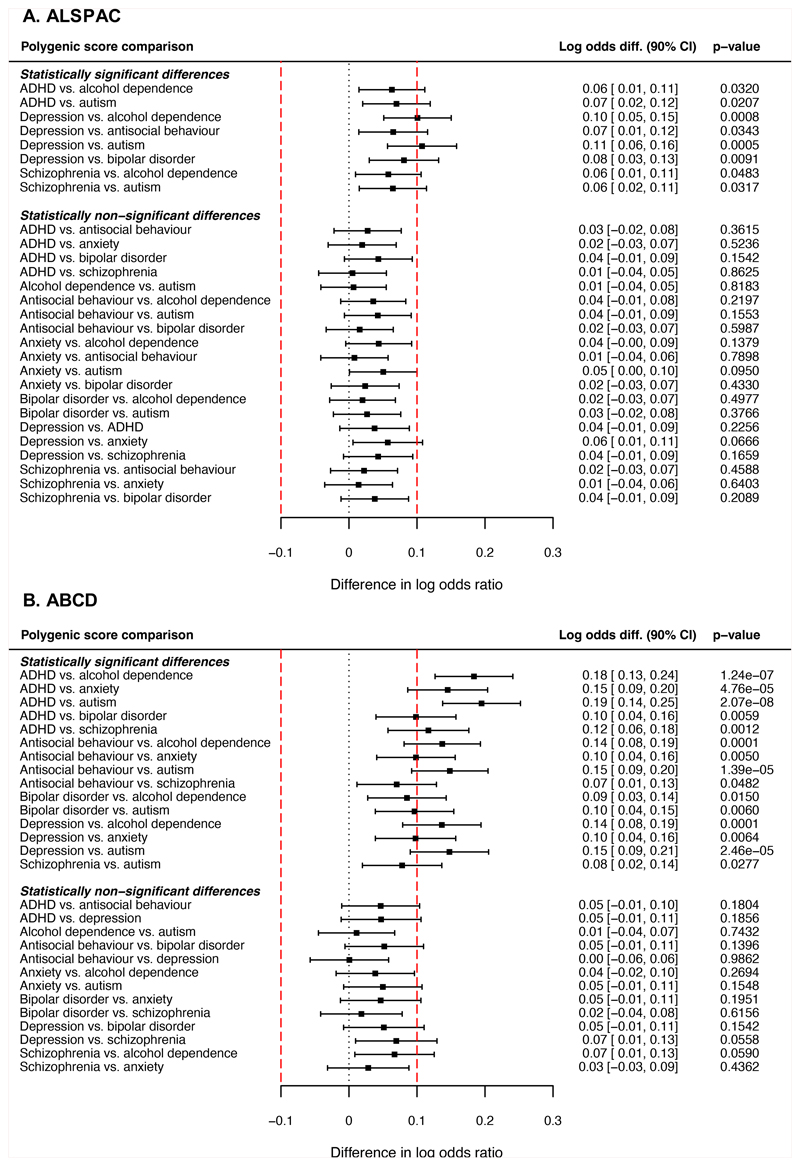
Pairwise differences between polygenic scores in their association with ACEs. Note: Data are presented as log odds differences +/- 90% CIs. Positive effect sizes reflect the first labelled polygenic score having a stronger positive average association with ACEs than the second polygenic score. Red dashed lines show the pre-specified equivalence bounds. 90% confidence intervals are presented and p-values are for the difference in log odds ratio between polygenic scores (two-sided tests). n=6,411 in ALSPAC and n=4,996 in ABCD.

Next, we examined whether polygenic scores for mental health problems differed in their average associations with ACEs. In ALSPAC, we found that polygenic scores for various mental health problems were differentially associated with ACEs (Wald-test *F* (7, 16,573)=2.62, p=0.011). Pairwise comparisons showed that the polygenic scores for depression, ADHD and schizophrenia predicted average risk of ACEs more strongly than various other polygenic scores (particularly for autism and alcohol dependence; Figure 3A). The 90% CIs for these differences did not fall entirely within the pre-specified equivalence bounds (-0.10 to 0.10 on the log odds scale; Figure 3A), suggesting that the differences were of a meaningful size.

#### ABCD

In the ABCD Study, polygenic scores for various mental health problems also showed different associations with ACEs (Wald-test *F* (7, 436,521)=7.68, p=2.60x10^-9^). Consistent with the ALSPAC findings, polygenic scores for depression, ADHD, and schizophrenia showed stronger average associations with ACEs than various other polygenic scores (particularly for autism and alcohol dependence; Figure 3B). However, in contrast to ALSPAC, polygenic scores for antisocial behaviour and bipolar disorder were more strongly associated with ACEs than some other polygenic scores (particularly autism and alcohol dependence). The 90% CIs for these differences did not fall within the equivalence bounds. Therefore, findings from both cohorts do not support the hypothesis that polygenic scores for different mental health problems would be equally associated with ACEs.

### 1c) Are some ACEs linked to greater polygenic risk for mental health problems than other ACEs?

#### ALSPAC

**Figure 4:**
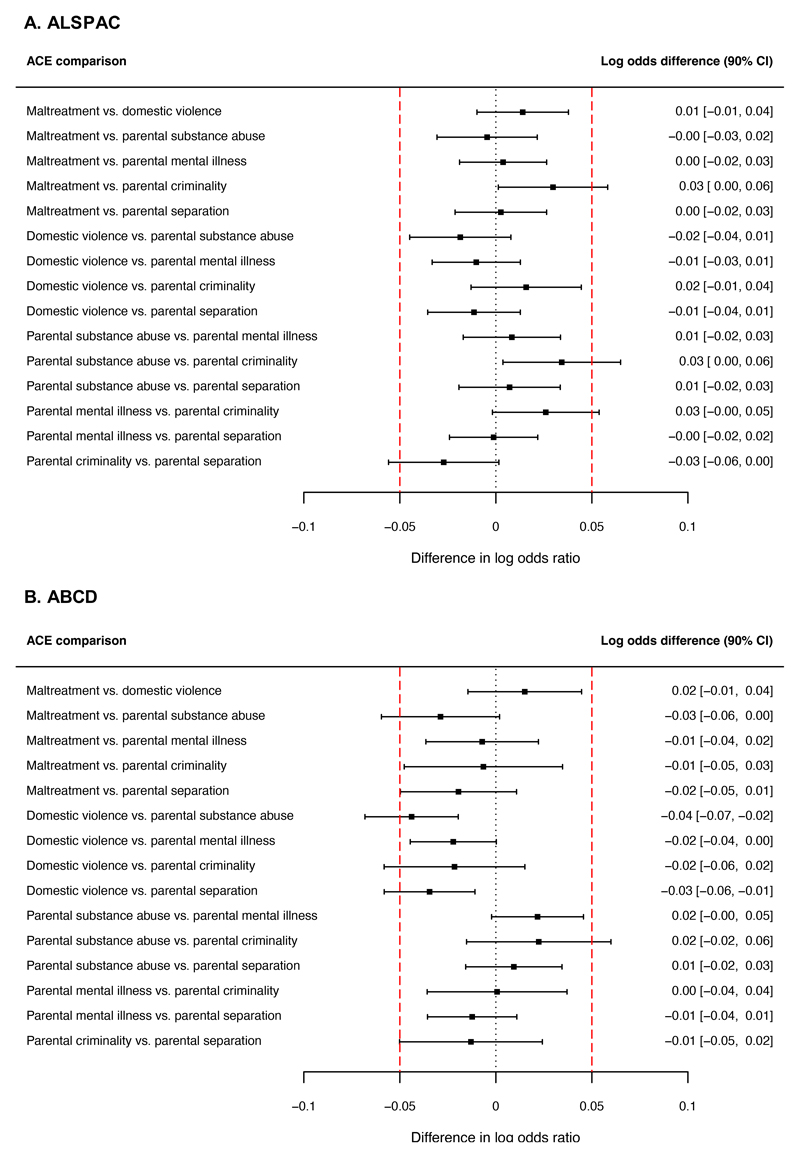
Pairwise differences between ACEs in their association with polygenic risk for mental health problems. Note: Data are presented as log odds differences +/- 90% CIs (two-sided tests). Positive effect sizes reflect the first labelled ACE having a stronger positive association with pooled polygenic risk for mental health problems; negative effect sizes reflect the second labelled ACE having a stronger positive association with pooled polygenic risk for mental health problems. The red dashed lines show the p re-specified equivalence bounds. n=6,411 in ALSPAC and n=4,996 in ABCD.

We next examined whether the associations between polygenic scores for mental health problems and ACEs differed across ACEs. There was no evidence to suggest that average polygenic risk for mental health problems differed across ACEs (Wald-test *F* (5, 5,319)=1.07, p=0.37). Furthermore, equivalence tests suggested that the majority of ACEs were associated with similar polygenic risk of mental health problems, as the 90% CIs for the differences between most ACEs fell inside the equivalence bounds (-0.05 to 0.05 on the log odds ratio scale; Figure 4A).

#### ABCD

Similar to ALSPAC, in the ABCD cohort, average polygenic risk for mental health problems was not significantly different across ACEs (Wald-test *F* (5, 246,200)=2.00, p=0.08). Equivalence tests also suggested that the majority of ACEs were associated with equal polygenic risk of mental health problems, as the 90% CIs for most differences between ACEs fell inside the equivalence bounds (Figure 4B). Therefore, findings from both cohorts did not support the hypothesis that parental mental illness, parental substance abuse, and parental criminality would be associated with higher polygenic risk for mental health problems than other ACEs.

### 2a) What proportions of the associations between ACEs with internalising and externalising problems are explained by observed polygenic scores for mental health problems?

#### ALSPAC

**Figure 5:**
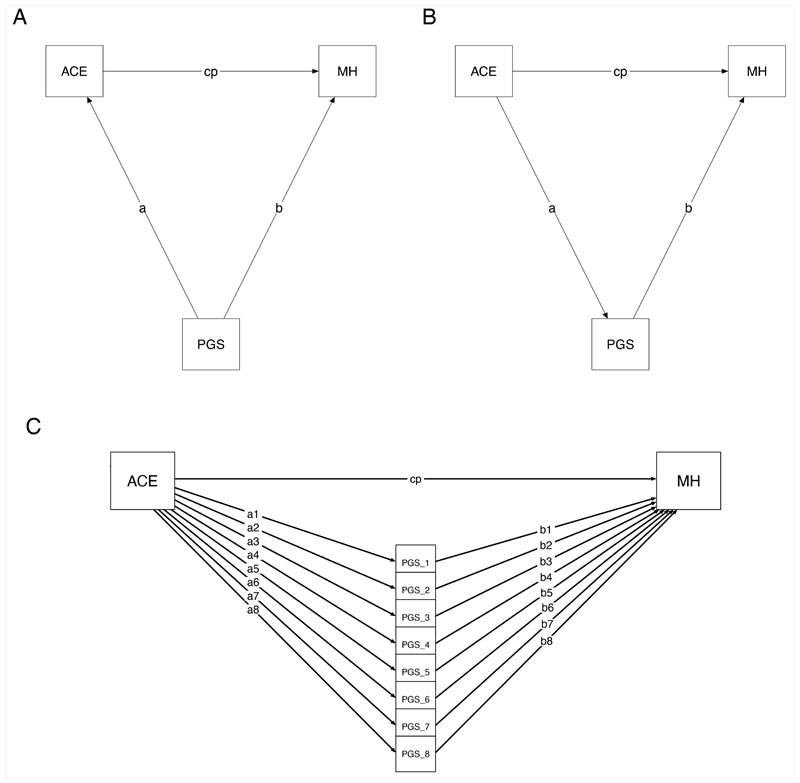
Diagrams showing structural equation models to estimate the genetic contribution to the associations between ACEs and mental health. Note. In all diagrams, ACE represents the adverse childhood experience, MH represents the mental health outcome (e.g., internalising problems or externalising problems) and PGS represents the polygenic score, with one polygenic score shown in panels A and B, and all 8 polygenic scores (PGS_1-PGS_8) shown in panel C. Panel A depicts the underlying conceptual model, in which the polygenic score is treated as a confou nder, whereas panel B depicts the statistical model to calculate the genetic confounding effect, in which the polygenic score is treated as a mediator. Note that conceptually, the polygenic score cannot be a mediator in the association between ACEs and mental health because genetic variants are set at conception and do not change throughout the lifespan. However, statistically, we can estimate the genetic confounding effect by treating the polygenic score as a mediator and calculating the indirect effect of ACEs on mental health through the polygenic score. Panel C represents the statistical model in which all 8 polygenic scores are included as mediators. Though not depicted in the figure to aid clarity, we will account for correlations between polygenic scores in the model.

**Figure 6:**
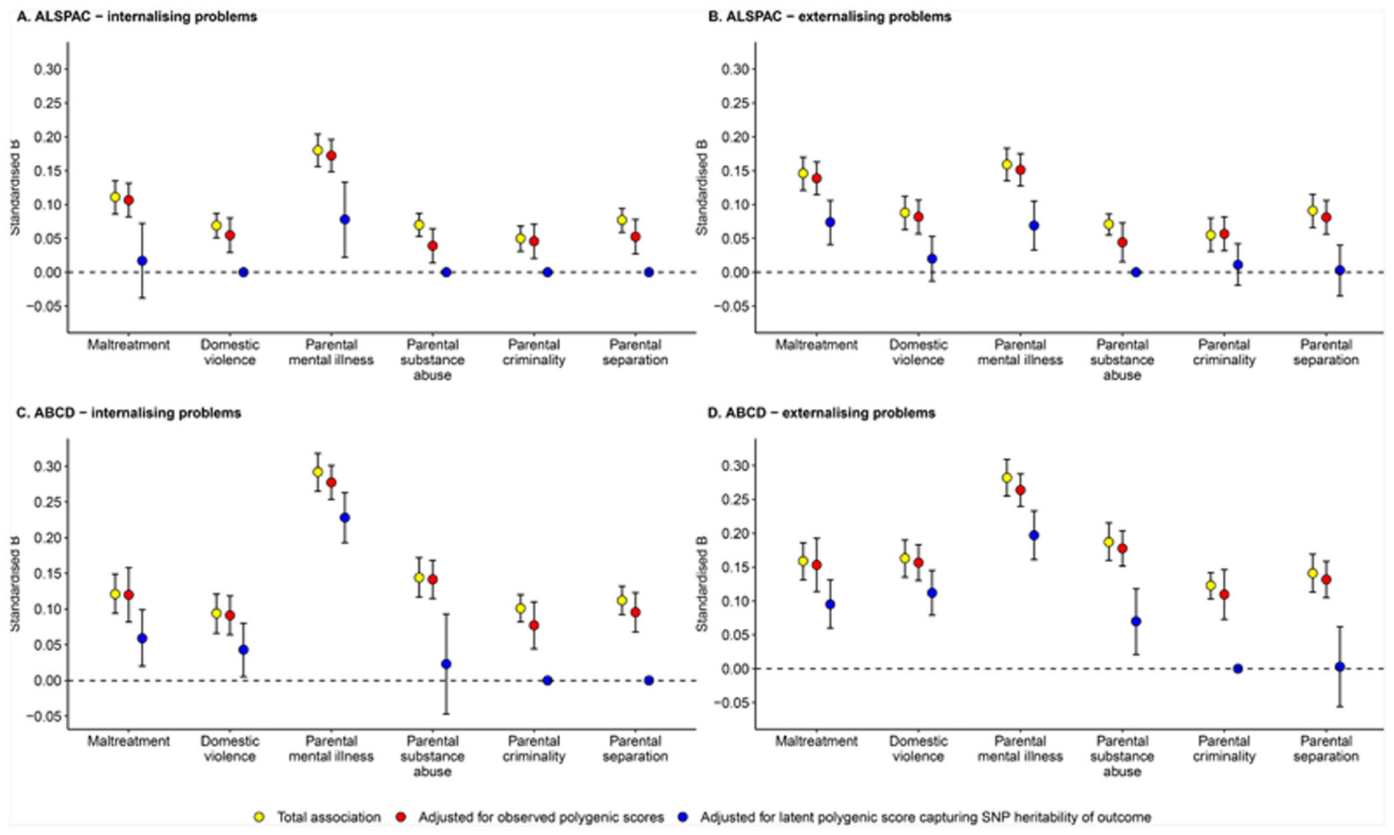
Genetic confounding of the associations between ACEs with internalising and externalising problems. Note. Data are presented as standardised beta coefficients +/- 95% CIs for associations between ACEs and mental health outcomes, before accounting for polygenic scores (yellow circles), and after accounting for (i) observed polygenic scores for mental health problems (red points), and (ii) a latent polygenic score capturing SNP heritability in the outcome (blue points). Panel A shows the associations between ACEs and internalising problems in ALSPAC; Panel B shows the associations between ACEs and externalising problems in ALSPAC; Panel C shows the associations between ACEs and internalising problems in ABCD; Panel D shows the associations between ACEs and externalising problems in ABCD. Tests were two-sided. Confidence intervals could not be reliably computed for associations attenuated to zero and therefore these estimates should be interpreted with caution. n=6,411 in ALSPAC and n=4,996 in ABCD.

To test genetic confounding, we next examined the proportion of the associations between ACEs and childhood mental health problems that were explained by polygenic scores for mental health problems (depression, anxiety, bipolar disorder, autism, ADHD, antisocial behaviour, alcohol use disorder, and schizophrenia) using a structural equation model (Figure 5C). In ALSPAC, polygenic scores for mental health problems explained a very small average proportion of the associations between ACEs and internalising problems at age 10 (4.4%, 95% CI=1.9-6.8%, p=0.0004). These polygenic scores also explained a small average proportion of the associations between ACEs and externalising problems at age 10 (5.8%, 95% CI=3.4-8.2%, p=3.18x10^-6^). Results for associations between specific ACEs with internalising and externalising problems are shown in Figure 6A-B (red points for adjusted associations) and Supplementary Table 2A. In contrast, negative control polygenic scores for handedness and cataracts did not explain any part of the associations between ACEs with internalising problems (average proportion=0.0%, 95% CI= -0.6;0.5, p=0.91) or externalising problems (average proportion= -0.1%, 95% CI= -0.5;0.4, p=0.77).

#### ABCD

Similar to ALSPAC, in the ABCD Study, polygenic scores for mental health problems explained a very small average proportion of the associations between ACEs and internalising problems at age 9/10 (3.0%, 95% CI=1.0-4.9%, p=0.003), and a small average proportion of the associations between ACEs and externalising problems at age 9/10 (5.0%, 95% CI=3.3-6.7%, p=6.38x10^-9^). Results for associations between specific ACEs with internalising and externalising problems are shown in Figure 6C-D (red points for adjusted associations) and Supplementary Table 2B. Negative control polygenic scores did not explain any of the associations between ACEs with internalising problems (average proportion=0.0%, 95% CI= -0.3%;0.4%, p=0.90) or externalising problems (average proportion=0.1%, 95% CI= -0.3%;0.5%, p=0.56). Taken together, these findings broadly support the hypothesis that observed polygenic scores account for a small proportion (defined as 5-20%) of the average association between ACEs and mental health problems, although the proportion captured for internalising problems was slightly smaller (<5%) than hypothesised.

### 2b) What proportions of the associations between ACEs with internalising and externalising problems are explained by latent polygenic scores capturing additional heritability in mental health problems?

Because polygenic scores for mental health problems only captured a very small proportion of variance in internalising problems (<1%) and externalising problems (1.6%; Supplementary Table 3), the previous analyses likely underestimated the magnitude of genetic confounding. To address this, we conducted a genetic sensitivity analysis^27^, which estimates genetic confounding using latent polygenic scores capturing SNP heritability in outcomes (6% and 9% for internalising and externalising problems, respectively^33,34^).

#### ALSPAC

In ALSPAC, the genetic sensitivity analysis suggested that a large average proportion of the associations between ACEs and internalising problems was explained by genetic confounding (90.3%, 95% CI=80.1-100%, p=1.76x10^-68^), with proportions ranging from 56.9% for parental mental illness to 100% for domestic violence, parental substance abuse, criminality, and separation (Supplementary Table 4A; Figure 6A [blue points for adjusted associations]). Similarly, a large average proportion of the associations between ACEs and externalising problems was accounted for by genetic confounding (76.5%, 95% CI=59.5%-93.6%, p=1.43x10^-18^), with proportions ranging from 49.4% for child maltreatment to 100% for parental substance abuse (Supplementary Table 4A; Figure 6B [blue points]). However, confidence intervals could not be reliably computed for some individual estimates (where the genetic confounding effect explained 100% of the associations; Supplementary Table 4; Figure 6A-B) and therefore such estimates should be interpreted with caution.

#### ABCD

In the ABCD Study, the genetic sensitivity analysis suggested that genetic confounding accounted for a large average proportion of the associations between ACEs and internalising problems (68.6%, 95% CI=55.5%-81.7%, p=1.07x10^-24^), with proportions ranging from 22% for parental mental illness to 100% for parental criminality and separation (Supplementary Table 4B; Figure 6C [blue points for adjusted associations]). Similarly, a large average proportion of the associations between ACEs and externalising problems was captured by genetic confounding (60.3%, 95% CI=48.7%-71.9%, p=2.22x10^-24^), with proportions ranging from 30.2% for parental mental illness to 100% for parental criminality (Supplementary Table 4B; Figure 6D [blue points]). These results indicate that the proportion of the associations between ACEs and mental health explained by genetic confounding is greater than the moderate amount (between 20% to 40%) hypothesised.

### Robustness analyses

To assess the robustness of our results, we conducted three sets of analyses. First, because reliable confidence intervals could not be computed for some results in the genetic sensitivity analysis (Supplementary Table 4), we were concerned that these results might have biased the pooled estimates of genetic confounding. We therefore re-estimated the average proportions of genetic confounding after excluding these results with unreliable confidence intervals. The average proportion of the associations between ACEs and internalising problems explained by genetic confounding was attenuated but still large (ALSPAC: 70.8%, 95% CI= 40.4-100%, p=4.88x10^-6^; ABCD: 52.9%, 95% CI=33.2-72.6%, p=1.33x10^-7^). This was also the case for the associations between ACEs and externalising problems (average proportion genetically confounded: 71.8% in ALSPAC [95% CI=51.4-92.3%, p=6.01x10^-12^] and 52.4% in ABCD [95% CI=38.5-66.3%, p=1.66x10^-13^]).

Second, we repeated all analyses in the complete case samples from ALSPAC and ABCD (N=4,106 and N=4,662, respectively) and observed largely consistent results (Supplementary Results 1).

Third, because we constructed the polygenic scores for bipolar disorder from an updated GWAS^35^ that differed from the older GWAS^36^ that we proposed to use in the Stage 1 pre-registration (Supplementary Table 5), we repeated the analyses with polygenic scores for bipolar disorder derived from the pre-registered GWAS. The results were consistent with the main findings (Supplementary Results 2).

## Discussion

This Registered Report examined the genetic contribution to the associations between adverse childhood experiences and mental health, in two prospective cohorts of over 11,000 children from the UK and US. Our findings provide insight into gene-environment correlations and genetic confounding of the relationship between ACEs and mental health.

With regard to gene-environment correlations, there are three key findings. First, children with higher polygenic scores for mental health problems had an elevated risk of ACEs. This gene-environment correlation was small but robust (replicating across cohorts) and negative control polygenic scores were not associated with ACEs. This supports our hypothesis and other (largely non-pre-registered) research showing that polygenic scores for mental health problems are associated with greater risk of exposure to childhood adversities^21–25,37,38^. Importantly, this does not suggest that exposure to ACEs is determined by genes, is the fault of the child, or is not preventable. Rather, the findings suggest that children with higher genetic liability to mental health problems are on average, slightly more likely to experience ACEs. However, ACEs are influenced by many factors (including social and environmental risks^39^) and can be effectively prevented through social interventions^40,41^.

Second, in both cohorts, polygenic scores for ADHD, depression, and schizophrenia were more strongly associated with risk of exposure to ACEs than some other polygenic scores (particularly alcohol use and autism). In the ABCD Study, polygenic scores for antisocial behaviour and bipolar disorder also showed stronger associations with ACEs. These results do not support our hypothesis that there would be no differences between polygenic scores, but broadly align with evidence showing that polygenic scores for ADHD, depression, and schizophrenia are independently associated with child maltreatment^37^ and bullying victimisation^23^, while polygenic scores for other psychiatric disorders are not. This finding should be interpreted with caution as it may reflect differences in predictive power of polygenic scores, given that the most predictive polygenic scores tended to be based on large GWAS samples and have higher SNP heritability (Supplementary Table 5). Alternatively, such differences might be because genetic liabilities to ADHD, depression, and schizophrenia have greater causal effects on exposure to ACEs than other genetic liabilities (e.g., because of stronger passive or evocative gene-environment correlations).

Third, different ACEs were associated with similar genetic risk of mental health problems in both cohorts. This was contrary to our hypothesis that parental mental illness, parental substance abuse, and parental criminality would be associated with greater (child) genetic risk of psychopathology than other ACEs, due to intergenerational genetic transmission. While these ACEs (originating in the parents) are likely to be linked to child genetic risk of psychopathology largely via passive gene-environment correlation, other ACEs might be related to genetic risk of psychopathology in part via evocative gene-environment correlation. Indeed, evidence suggests that children at genetic risk for externalising problems are more likely to experience negative parenting via evocative gene-environment correlation^16,17^, and evocative gene-environment correlations were found to partly underlie risk of maltreatment^42^. Importantly, evidence of such evocative gene-environment correlation does not mean children are to blame for ACEs – rather, parents are responsible for protecting them and reacting to their behaviour in an appropriate way^42^. Evidence of evocative gene-environment correlation would therefore highlight the importance of family-based interventions to help parents respond effectively to their child’s behaviour, and support children with vulnerabilities.

With regard to genetic confounding, we first found that observed polygenic scores for mental health problems explained on average, 3-5% of the associations between ACEs and internalising problems and 5-6% of the associations between ACEs and externalising problems. In contrast, negative control polygenic scores did not account for any of the associations between ACEs and mental health problems. These results broadly support our hypothesis that a small proportion (defined as 5-20%) of the associations between ACEs and mental health would be captured by polygenic scores for psychopathology. However, these results likely under-estimate the magnitude of genetic confounding as the polygenic scores for mental health problems only captured a very small amount of variation (<1% and <1.6%, respectively) in internalising and externalising outcomes.

To address this, we conducted a genetic sensitivity analysis^27^ using latent polygenic scores capturing SNP heritability in internalising and externalising problems (6% and 9%, respectively). This analysis suggested that genetic confounding accounted for a large average proportion of the associations between ACEs with internalising and externalising problems, in both cohorts. However, we caution against drawing strong conclusions based on the specific proportions of genetic confounding, for three reasons. First, the precise magnitude of genetic confounding varied between cohorts, and point estimates were greater in ALSPAC than in the ABCD Study. This is likely to be because ACEs had weaker associations with mental health problems in ALSPAC (Figure 5), increasing the likelihood that genetic confounding could account for the association. In contrast, the magnitude of associations between polygenic scores and ACEs did not differ between both cohorts (Supplementary Table 3). Second, confidence intervals could not be reliably estimated for some specific estimates of genetic confounding, in particular for proportions of 100% (largely observed for internalising outcomes in ALSPAC), suggesting that these proportions may not be reliable. Third, the genetic sensitivity analysis is best suited for scenarios in which the polygenic score strongly and specifically predicts the outcome^27^. Given the lack of available GWASs for both child internalising and externalising problems, we used polygenic scores for adult psychiatric disorders, which showed similar or stronger magnitude associations with ACEs as with child internalising and externalising problems (Supplementary Table 3). The use of a polygenic score that is not specific to the outcome may result in overestimated genetic confounding (discussed in detail in the Supplementary Discussion). Therefore, it will be important to repeat the genetic sensitivity analysis with future GWASs of child internalising and externalising problems, when available.

Despite our cautious interpretation surrounding specific estimates of genetic confounding, the overall pattern of results supports findings from other genetically informed designs, with different assumptions and sources of bias. For example, we found that child maltreatment was largely associated with internalising and externalising problems, independent of genetic confounding. This is consistent with evidence of causal effects of maltreatment on psychopathology from Mendelian Randomisation^42^, co-twin control^43^, and other quasi-experimental studies^44^. We also found that parental mental illness was associated with internalising and externalising problems independent of genetic confounding, which supports evidence from Children-of-Twins (CoT) and adoption studies^45–47^. In contrast, we found that parental substance abuse, parental criminality, and parental separation were predominantly associated with internalising and externalising problems via genetic confounding. Notably, similar genetically confounded associations with psychopathology have also been reported for parental substance abuse in CoT^48,49^ and adoption studies^50^, for parental criminality in an adoption study^51^, and for parental separation in some^52^ (though not all^53^) CoT studies.

We acknowledge some limitations. First, it is possible that observed associations might be inflated by reporting bias, as parents with genetic liability to psychopathology might be more likely to perceive ACEs^54^ and child psychopathology, as well as transmit genetic liability to their children. Future studies using different informants to measure ACEs and psychopathology (e.g., from objective records to more subjective self-reports) are needed to map the impact of reporting biases on observed gene-environment correlations^38,55^ and estimates of genetic confounding. Second, ALSPAC and the ABCD Study differed in various ways, such as the country of origin (UK vs USA), historical context (born in 1991-1992 vs 2006-2008), and prevalence of ACEs (e.g., higher rates of maltreatment and parental criminality in ALSPAC, perhaps due to repeated assessments [vs a single assessment in ABCD]). The ABCD analysis is therefore not a direct replication of the ALSPAC findings, and any differences in findings might be attributable to these cohort differences. However, the overall pattern of results was consistent across both cohorts, indicating that the findings are robust. Third, as discussed, it was not possible to infer whether differential associations between polygenic scores for psychiatric disorders and ACEs reflected specific genetic liabilities underlying risk of ACEs, or differences in the predictive power of polygenic scores (e.g., due to different GWAS discovery sample sizes). Fourth, our analysis was limited to individuals of European descent to match the ancestry of the GWAS discovery samples^56^. Once large-scale trans-ancestry GWASs become available, it will be important to replicate our findings in ancestrally diverse samples, to ensure greater representation in research^57^. Finally, these findings reflect average population effects, and do not preclude the existence of causal effects of certain ACEs (e.g., parental substance abuse, parental criminality, and parental separation) on child psychopathology in subpopulations.

Our findings have implications for future research. First, to understand the extent to which the observed gene-environment correlations are passive or evocative in nature, future studies should integrate polygenic scores into family-based designs (e.g., parent-offspring trios)^30^. Second, to the extent that ACEs are causal risk factors for psychiatric disorders, genetic variants influencing exposure to ACEs (i.e. gene environment correlations) might be captured in GWASs of those disorders^55,58^. If GWASs of ACEs were to become available, future genetically informed studies could test whether this reflects one of the origins of the observed associations between polygenic scores for psychiatric disorders and ACEs. Third, the gene-environment correlations observed here challenge the assumption in gene-environment interaction (GxE) studies that genetic influences on psychopathology and ACEs are independent^13,59^. Future GxE studies on childhood adversity and psychopathology should adopt methods that account for such gene-environment correlations to mitigate bias^13,59^. Lastly, this study suggests that non-genetically informative studies are likely to have overestimate the causal contribution of ACEs to mental health problems. To provide accurate estimates on the causal effects of ACEs, future studies should employ methods that account for genetic confounding, and triangulate evidence across methods with different assumptions and sources of potential bias^60,61^. More broadly, combining genetically informed methods with open science practices (e.g., pre-registration / Registered Reports) will help to address multiple sources of bias (e.g., genetic confounding, researcher bias^62^, and publication bias^63^) to enable rigorous evidence on the effects of ACEs on health.

Our findings also have implications for interventions. Because child maltreatment and parental mental illness were largely associated with child psychopathology independent of genetic influences, preventing these ACEs may not only improve child welfare and family functioning, but may also help to prevent child psychopathology in the population. Such interventions could include parenting support programmes to prevent maltreatment^40^, and more accessible psychiatric treatment for parents with mental health problems. In contrast, preventing ACEs with entirely genetically confounded effects is unlikely to substantially impact child psychopathology at the population level, although such interventions are likely to have other important positive outcomes (e.g., for child welfare, family functioning, and potentially physical health^64–67^). Furthermore, because polygenic scores for mental health problems accounted for at least part of the associations between all ACEs and psychopathology, strategies that address heritable psychiatric vulnerabilities in children exposed to ACEs (e.g., through skills building^68^ or fostering positive family interaction) should reduce their risk of developing psychopathology.

## Methods

### Change in replication cohort

As stated in our Stage 1 protocol (https://doi.org/10.6084/m9.figshare.13580777.v1), this Registered Report originally proposed to replicate findings from ALSPAC in the Child and Adolescent Twin Study in Sweden (CATSS) dataset, and not the ABCD Study. However, after receiving Stage 1 in-principle acceptance, we experienced two unforeseen issues which meant that we could not use the CATSS dataset: (1) data could not be accessed in a timely manner because of covid-related travel restrictions for Sweden, and (2) data access restrictions from the Swedish National Board of Health and Welfare meant that we could not use national registry data to measure ACEs, as originally proposed. We therefore proposed and received permission to use the ABCD Study as an alternative replication sample to CATSS (after peer review of the protocol for analysis on ABCD). Importantly, we had not accessed data from either CATSS or the ABCD Study at the time in which we proposed to use the ABCD Study, so we were blind to the results in these cohorts (though we had undertaken analysis in ALSPAC). To provide transparency about what we intended to do in the Stage 1 protocol, we report all details about the CATSS dataset in Supplementary Methods 1.

### Ethics information

Ethics approval for ALSPAC was obtained from the ALSPAC Ethics and Law Committee and the Local Research Ethics Committees. Informed consent for the use of data collected via questionnaires and clinics was obtained from participants following the recommendations of the ALSPAC Ethics and Law Committee at the time. Consent for biological samples has been collected in accordance with the Human Tissue Act (2004). Ethics approval for the ABCD Study was given by a central Institutional Review Board (IRB) at the University of California, San Diego, and in some cases by individual site IRBs (e.g. Washington University in St. Louis)^69^. Parents or guardians provided written informed consent after the procedures had been fully explained and children assented before participation in the study^70^.

### Design

ALSPAC and the ABCD Study are prospective longitudinal cohort studies. A description of these datasets and their measures is below.

### The Avon Longitudinal Study of Parents and Children

#### Sample

The Avon Longitudinal Study of Parents and Children (ALSPAC) is a longitudinal study of children born in the United Kingdom in 1991-1992. ALSPAC sought to recruit all pregnant women in the former county of Avon, United Kingdom, with an expected due date between April 1, 1991 and December 31, 1992. The initial sample consisted of children of 14,541 mothers. Children have been followed-up and assessed repeatedly across development through questionnaires, face-to-face interviews and physical and psychological assessments (including biological assays)^71–73^. The study website contains details of all the data that is available through a fully searchable data dictionary and variable search tool (http://www.bristol.ac.uk/alspac/researchers/our-data/). 49% of the analytic sample was female.

#### Measures

##### Adverse childhood experiences

We examined six ACEs: maltreatment, domestic violence, parental mental illness, parental substance abuse, parental separation, and parental criminality. These experiences all involve adversity in the family context, and were included in the Centers for Disease Control and Prevention Adverse Childhood Experiences Study^3,74^ and the World Health Organization ACE international questionnaire^75^. In ALSPAC, these ACEs were assessed prospectively through parent and child reports via questionnaires at multiple assessment phases from birth to age 9 years (115 months). Details of these assessments are provided in Supplementary Table 6. We derived binary measures reflecting exposure to each ACE according to definitions shown in Supplementary Table 6 and recommended by a previous ALSPAC Data Note on ACE measures^76^. Note that sub-types of maltreatment (physical, sexual, and emotional abuse, and neglect) were combined into a single measure due to low individual prevalence and high co-occurrence^76,77^. Measures of each ACE were derived for participants with responses to ≥50% of the questions assessing that ACE between birth to age 9 years. We used multiple imputation to estimate ACE exposure in participants with responses to <50% but ≥10% of questions assessing the ACE (see Supplementary Methods 2 for further details of the multiple imputation procedure).

##### Mental health problems

Internalising problems and externalising problems were assessed through parent reports on the Development and Wellbeing Assessment (DAWBA)^78^ at age 10 years. The DAWBA is a semi-structured interview assessing multiple domains of child psychopathology with good validity^78^ and reliability^79^. Items from the DAWBA used to derive the mental health measures are presented in Supplementary Table 7.

*Internalising problems* were assessed through modules on separation anxiety (11 items, scale from 0-20), social anxiety (6 items, scale from 0-12), generalised anxiety (15 items, scale from 0-28), and major depression (15 items, scale from 0-15). We derived one overall measure of internalising problems through the following steps. First, we calculated the mean for each of the four modules (separation anxiety, social anxiety, general anxiety, and major depression) for participants with data for ≥50% of the items, before standardising the scores. Next, we summed the scores across the anxiety sub-scores and standardised the measure, so we have one overall measure of anxiety, and one for major depression. Last, we summed these anxiety and depression scores, before standardising the overall single measure.

*Externalising problems* were assessed through modules on hyperkinesis/ADHD (18 items, scale from 0-36) and conduct/oppositional disorders (17 items, scale from 0-34). To derive one overall measure of externalising problems, we first calculated the mean for each of the two modules for participants with data for ≥50% of the items. We then standardised the two scores and summed them, before standardising the overall single measure.

##### Genotyping and quality control

ALSPAC children have been genotyped using the Illumina HumanHap550 quad chip genotyping platforms by 23andme subcontracting the Wellcome Trust Sanger Institute, Cambridge, UK and the Laboratory Corporation of America, Burlington, NC, US. Quality control (QC) was carried out in PLINK^80^, adhering to standard guidelines^81,82^ which have been previously used effectively for analysis of genetic data in ALSPAC^21,23,83^. Specifically, samples were removed on the basis of (1) low call rate (poor DNA quality), (2) outlying heterozygosity across autosomes, (3) relatedness (based on identity-by-state), (4) gender mismatches, and (5) non-European population ancestry. SNPs were removed on the basis of (1) low call rate, (2) extreme deviation from Hardy-Weinberg equilibrium, and (3) low minor allele frequency. Further details are provided in Supplementary Table 8.

##### Polygenic scores

We derived polygenic scores for mental health problems; namely, major depressive disorder, anxiety disorder, bipolar disorder, autism, ADHD, antisocial behaviour, alcohol use disorder, and schizophrenia. We selected these polygenic scores because they (i) index genetic liability to a range of mental health problems, and (ii) have been found to be associated with ACEs^21–23,26^ and/or psychopathology in young people^84–87^. We also derived negative control polygenic scores for traits with no known association with ACEs or mental health (namely, handedness and cataracts). All polygenic scores were generated using GWAS summary statistics which (i) were derived from European samples that did not include ALSPAC and ABCD participants (to avoid sample overlap), and (ii) had N > 16,000 in the discovery sample (to ensure adequate power). Supplementary Table 5 provides details of the GWAS summary statistics which were used to derive polygenic scores.

In our Stage 1 protocol, we specified that if new, larger GWASs were published after submission, we would use the updated summary statistics to benefit from greater power (and report any such changes in the Stage 2 submission). Since the Stage 1 submission, new, larger GWASs were published for bipolar disorder^35^ (N=413,466 versus N=51,710 in the original GWAS^36^) and antisocial behaviour problems^88^ (N=83,674 versus N=16,400 in the original GWAS^87^), and so we derived polygenic scores from these updated summary statistics for our main analyses. For transparency, we also report the results using the originally pre-registered GWAS summary statistics^36^ to derive the polygenic score for bipolar disorder. We did not do this using the older GWAS for antisocial behaviour^87^, as we realised that there was sample overlap for ALSPAC, which could have led to biased estimates^89^.

Polygenic scores were derived in PRSice software^90,91^, using the following method: First, SNPs from participants were matched with SNPs reported in the GWAS summary statistics for each phenotype (e.g., each mental health problem). Clumping was conducted to remove SNPs in linkage disequilibrium (r^2^>0.1 within a 250–base pair window). Next, we summed the alleles associated with the phenotype and weighted them by their effect sizes reporting the corresponding GWAS, to compute polygenic scores. We included all matched SNPs regardless of the nominal significance for their association with ACEs. To control for population stratification, we residualised polygenic scores for the first 10 principal components estimated from the genome-wide SNP data. To facilitate interpretability, all polygenic scores were standardised to have a mean of 0 and standard deviation of 1.

### The Adolescent Brain and Cognitive Development (ABCD) Study

#### Sample

The Adolescent Brain and Cognitive Development (ABCD) Study is a prospective cohort of 11,878 children born during the period 2006-2008, and their parents from 21 sites in the United States. The 21 geographic locations of the ABCD research sites are nationally distributed and generally represent the range of demographic and socio-economic diversity of the U.S. birth cohorts comprising the ABCD study population^92^. Full details on the recruitment strategy are available elsewhere^93^. Briefly, children aged 9-10 years were recruited through probability sampling of public and private elementary schools within the catchment areas of the 21 research sites. School selection was based on gender, race and ethnicity, socioeconomic status, and urbanicity. Inclusion criteria were the child’s age and attending a public or private elementary school within the catchment areas. Exclusion criteria for children were limited to not being fluent in English, having a parent not fluent in English or Spanish, major medical or neurological conditions, gestational age <28 weeks or birthweight <1200 g, contraindications to MRI scanning, a history of traumatic brain injury, a current diagnosis of moderate/severe autism spectrum disorder, intellectual disability, schizophrenia, or alcohol/substance use disorder^94^. Assessments were made through in-person visits. This study used data from the baseline assessment (ages 9-10) and 1-year follow-up (ages 10-11), from ABCD Data Release 3.0. 47% of the analytic sample was female.

#### Measures

##### Adverse childhood experiences

Consistent with the ALSPAC cohort, we assessed six ACEs (maltreatment, domestic violence, parental mental illness, parental substance abuse, parental separation, and parental criminality) between birth and age 9-10 years. These ACEs have been assessed through parent and child reports from validated questionnaires at the baseline and 1-year follow-up assessment^95^. Details of these assessments are reported in Supplementary Table 9. In brief, maltreatment was assessed using the parent-reported Kiddie-Structured Assessment for Affective Disorders and Schizophrenia module for post-traumatic stress disorder^96,97^ (KSADS-PTSD; with 8 items for physical, sexual, and emotional abuse) and the Children’s Report of Parental Behavioral Inventory^98^ (with 5 items for neglect), consistent with previous studies^42^. Domestic violence was assessed using parent reports on the KSADS-PTSD, and parent and child reports on the Family Environment Scale – Family Conflict Subscale^99,100^. Parental mental illness and substance abuse were assessed via parent reports on the Family History Assessment Module^101^ and the Adult Self Report^102,103^. Parental criminality was assessed through parent reports on the Adverse Life Events Scale^104^, and parental separation was assessed through parent reports on the Demographic Survey. Measures of each ACE were derived for participants with responses to ≥50% of the questions assessing that ACE between birth to age 9-10 years.

##### Mental health problems

Internalising problems and externalising problems were assessed using parent reports on the Child Behavior Checklist (CBCL)^105^ from the baseline assessment at age 9/10. The CBCL is a 119-item, 3-point scale questionnaire which measures problems occurring in the past 6 months, with excellent reliability and validity^106^. Items from the CBCL used to derive the mental health measures are presented in Supplementary Table 10.

*Internalising problems* were assessed through the anxious/depressed, withdrawn/depressed, and somatic complaints subscales (32 items), as recommended^107^. *Externalising problems* were assessed through the rule-breaking behaviour, aggressive behaviour, and attention problems subscales (45 items). These subscales broadly map onto the DAWBA subscales used to assess internalising and externalising problems in ALSPAC, maximising consistency between the samples. To derive composite scores of internalising and externalising problems, we summed scores across the relevant items (for participants with data for >50% of the items) before standardising the summary measures.

##### Genotyping and QC

Children from the ABCD Study have been genotyped from blood and saliva samples using the Affymetrix NIDA SmokeScreen Array^108^. Sample preparation and genotyping was performed by Rutgers RUCDR. Initial QC was performed by the ABCD Data Analysis, Informatics & Resource Center following the Ricopili pipeline^109^ (see Supplementary Table 8 for details). Imputation was then performed on genotype data using the TOPMed imputation server, following pre-imputation steps instructed at: https://topmedimpute.readthedocs.io/en/latest/prepare-your-data/. In line with previous ABCD studies^42,110,111^, we performed additional QC on the imputed genetic data (Supplementary Table 8), including removing samples with high relatedness and non-European population ancestry, and removing SNPs which deviate from Hardy-Weinberg equilibrium, have a low minor allele frequency, and poor imputation quality.

##### Polygenic scores for mental health problems

We derived polygenic scores for mental health problems and negative controls using the same procedure as described for ALSPAC participants. We also residualised polygenic scores for genotyping batch as ABCD participants have been genotyped in multiple batches.

### Analysis plan

We conducted all statistical analyses in R Version 3.6.2^112^, focusing first on the ALSPAC cohort before testing whether the findings replicate in the ABCD Study (originally planned to be the CATSS dataset). Below we describe the statistical analyses that we will use to test each of our aims and hypotheses (summarised in Table 1). The multiple imputation procedure for ALSPAC and ABCD data is described in the Supplementary Methods 2-3.

#### Aim 1: Investigate whether children with genetic liability to mental health problems are more likely to be exposed to ACEs

##### Hypothesis 1a

We first tested the associations between polygenic scores for mental health problems and ACEs through logistic regression models. We ran separate models for each ACE and each polygenic score (including negative controls). Log odds coefficients were exponentiated to obtain odds ratios reflecting odds of exposure to each ACE per one standard deviation increase in the polygenic score. These models (and all further analyses) controlled for sex and were two-sided. To account for multiple testing, we computed false discovery rate corrected *p*-values^113^.

In order to obtain a single effect size reflecting the average association between polygenic scores for mental health problems and ACEs, we pooled the results across all logistic regression models within each cohort. This procedure was performed using the ‘agg’ function in the *MAd* package^114^, which accounts for correlations across effect sizes (as a function of the same sample). We pooled two sets of results: 1) for associations between polygenic scores for mental health problems and ACEs, and 2) for associations between negative control polygenic scores and ACEs.

Because null hypothesis significance testing cannot enable substantive interpretation of statistically non-significant findings, we conducted an equivalence test^115^ to quantify support for the null hypothesis. This involves assessing whether the 90% confidence intervals for the effect size lie entirely inside pre-specified equivalence bounds indexing the smallest effect size of interest. If the confidence intervals lie inside the equivalence bounds, the effect size can be said to be no more than trivially small. If the confidence intervals are not inside the equivalence bounds, the effect size can be said to be of meaningful magnitude. Note that the 90% (rather than 95%) confidence intervals are used, corresponding to (1–2α) × 100%, because the effect size is tested against two equivalence bounds separately (i.e., the upper and lower bound).

To select equivalence bounds, we followed guidance to use the lower confidence interval of a meta-analytic estimate of the effect of interest^115,116^. Because no such meta-analysis exists, we conducted a meta-analysis of all studies^21–26^ that to our knowledge, have tested the association between polygenic scores for mental health problems (see https://osf.io/2uc4p/?view_only=2d9afc1b072b4507ba11ba8771aaab62 for code and results). The pooled association between polygenic scores for mental health problems and ACEs was OR=1.10 (95% CI=1.06-1.14). We thus selected equivalence bounds of 0.94-1.06 on the odds ratio scale, because 1.06 was the lower confidence interval of the meta-analytic effect and 0.94 is the equal delta of 1.06 in the opposite direction on the log odds ratio scale.

We proposed to infer support for Hypothesis 1a (that children with greater genetic liability to mental health problems would have a higher risk of experiencing ACEs) if 1) the pooled odds ratio for the association between polygenic scores for mental health problems and ACEs was greater than 1 and statistically significant, 2) the 90% confidence interval for this effect was not within the equivalence bounds, and 3) the pooled odds ratio for the association between negative control polygenic scores and ACEs was non-significant. The interpretation of alternative patterns of results is shown in Table 1.

##### Hypothesis 1b

We next tested whether polygenic scores for certain mental health problems are more strongly associated with ACEs than other polygenic scores. To do so, we first used a structural equation model to estimate the associations between each polygenic score and each ACE (Supplementary Fig. 1). This model accounted for correlations between polygenic scores, allowing us to estimate the independent effect of each polygenic score on each ACE. From the model, we calculated the average effect of each polygenic score across all ACEs, estimated as: $\frac{\left(a1+a2+...+a6\right)}{6}$ for the first polygenic score (“PGS_1” in Supplementary Fig. 1) $\frac{\left(b1+b2+...+b6\right)}{6}$ for the second polygenic score (“PGS_2” in Supplementary Fig. 1), and so forth for each polygenic score. These analyses were conducted using the *lavaan* package^117^, using the WLSMV estimator with robust standard errors, and the ‘ordered’ argument (for the binary ACE endogenous variables). To aid interpretation, we converted the resulting probit coefficients into odds ratios using the formula: *exp*(probit $\hat{\beta }$ × 1.8)^118,119^. We then conducted a Wald test (using the “lavTestWald” function) to test whether the average effect of each polygenic score on all ACEs varied across polygenic scores. If the Wald test was statistically significant (p<0.05), we conducted pairwise comparisons to assess which polygenic scores differ in prediction of ACEs.

Lastly, we tested for statistical equivalence between different polygenic scores in their average association with ACEs by (1) calculating differences in the average effects of polygenic scores, expressed as (log) odds ratios^120^, and (2) assessing whether 90% confidence intervals for these differences fall within equivalence bounds of -0.10 to 0.10. We selected these equivalence bounds by identifying the smallest effect size that we have 95% power to detect (log odds difference = 0.10, 95% CI=0.07-0.13). This approach is recommended in the absence of a strong theoretical justification for equivalence bounds^115^, which was the case as no previous study has formally tested differences between polygenic scores in the association with ACEs.

We proposed to infer support for Hypothesis 1b (that polygenic scores for different mental health problems would equally predict exposure to ACEs) if the Wald test was statistically non-significant (*p*>0.05) and the 90% confidence intervals for the differences between polygenic scores (in their associations with ACEs) fell within the equivalence bounds. The interpretation of alternative patterns of results is shown in Table 1.

##### Hypothesis 1c

Next, we tested whether some ACEs were associated with higher polygenic risk of mental health problems than other ACEs. To do so, we used the same structural equation model as estimated for Hypothesis 1b (shown in Supplementary Fig. 1), and calculated the average effect of all polygenic scores for mental health problems on each ACE, estimated as: $\frac{\left(a1+b1+...+h1\right)}{8}$ for the first ACE (“ACE_1”), $\frac{\left(a1+b2+...+h2\right)}{8}$ for the second ACE (“ACE_2”), and so forth for each ACE. We converted results to odds ratios using the formula: *exp*(probit $\hat{\beta }$ × 1.8)^118,119^. We then used a Wald test to test whether the average effect of all polygenic scores for mental health problems on each ACE varies across ACEs. Lastly, we tested for statistical equivalence between different ACEs in their association with polygenic scores by (1) calculating differences in (log) odds ratios between ACEs, and (2) assessing whether 90% confidence intervals for these differences fall within equivalence bounds of -0.05 to 0.05. We selected these equivalence bounds because 0.05 is the smallest effect size that we have 95% power to detect (log odds difference = 0.05, 95% CI=0.03-0.07). We adopted this approach in the absence of theoretical justification for equivalence bounds^115^, as no previous study has tested for differences between ACEs in their association with polygenic scores for psychopathology.

We proposed to infer support for Hypothesis 1c (that parental mental illness and parental substance abuse would be associated with higher polygenic risk for mental health problems) if 1) the Wald test was significant (*p*<0.05) and further pairwise comparisons (between parental mental illness, parental substance abuse, and parental criminality with all other ACEs) showed that these ACEs were associated with higher polygenic risk than other ACEs, and 2) the 90% confidence intervals for these differences were not within the equivalence bounds. Interpretation of alternative patterns of results is shown in Table 1.

#### Aim 2: Investigate the extent to which genetic liability explains the associations between ACEs and mental health

##### Hypothesis 2a

To test the proportion of the associations between ACEs and mental health (internalising and externalising problems) explained by observed polygenic scores, we used structural equation models in the *lavaan*^117^ package. Fig. 5 depicts these models, with panel A showing the underlying conceptual model, panel B showing the statistical model with one polygenic score, and panel C showing the statistical model with multiple polygenic scores. As shown in panels B and C, polygenic scores were treated as mediators, as mediation and confounding are statistically equivalent^121^. The genetic confounding effect was therefore calculated as the indirect effect of the ACE on mental health through the polygenic scores: (a1 * b1) + (a2 * b2) + … + (a8 * b8), based on Fig. 5C. Notably, this estimate does not conflate genetic confounding with genetic effects on mental health mediated via exposure to ACEs (see ^27^ and https://osf.io/2uc4p/?view_only=2d9afc1b072b4507ba11ba8771aaab62 for further explanation and simulations demonstrating this). In turn, the proportion of the association between the ACE and mental health outcome explained by the polygenic scores was calculated as: $\frac{\left(al*b1\right)+\left(a2*b2\right)+\cdot \cdot \cdot +\left(a8*b8\right)}{\left(a1*b1\right)+\left(a2*b2\right)+\cdot \cdot \cdot +\left(a8*b8+\text{cp}\right)}.$

For this analysis, we included all polygenic scores (i.e., 8 mediators) and estimated separate models for each ACE and each mental health outcome (internalising and externalising problems). As a quality control check, we estimated a separate model including only negative control polygenic scores (Supplementary Figure 1).

To obtain a single estimate reflecting the proportion of the associations between ACEs and mental health outcomes captured by observed polygenic scores, we averaged the results across 6 models for all ACEs (for internalising and externalising problems, separately). This was performed using the ‘agg’ function from the *MAd* package^114^. Prior to aggregating the results, we planned to transform proportions using the Freeman-Tukey double arcsine transformation^122^ to normalise and stabilise the variance of the sampling distribution. However, it was not possible to apply this transformation across the results as several proportions were less than zero – which can arise when the direct and indirect effects are in different directions. We therefore used the raw proportions for consistency across all models. We pooled two sets of results, reflecting proportions of the associations between ACEs and mental health captured by: 1) polygenic scores for mental health problems, and 2) negative control polygenic scores.

We proposed to infer support for Hypothesis 2a (that a small proportion of the associations between ACEs and mental health problems would be explained by polygenic scores) if 1) polygenic scores for mental health problems explained, on average, between 5% to 20% of the associations, and 2) the average proportion of the association explained by negative control polygenic scores was not significantly different from zero. We proposed to interpret alternative proportions of less than 5% as “very small”, proportions between 20% and 40% as “moderate”, and proportions of more than 40% as “large”, broadly in line with guidance for interpreting effect sizes^123^.

##### Hypothesis 2b

Lastly, we estimated the proportion of the associations between ACEs and mental health problems explained by a latent polygenic score which captures SNP heritability in the mental health outcome. This genetic sensitivity analysis^27,124^ involves estimating the structural equation model shown in Fig. 5B from a correlation matrix. This matrix includes correlations between the polygenic score and the ACE (*a* path), the polygenic score and the mental health outcome (*b* path), and the ACE and the mental health outcome (*cp* path). Critically, this correlation matrix can be modified to reflect additional genetic variance captured in the outcome. For example, as the SNP-based heritability of parent-reported childhood internalising problems is 6%^33^, the correlation coefficient from the polygenic score to internalising problems (*b* path) can be changed to *r* = 0.24 (calculated by taking the square-root of 0.06). The correlation coefficient for the *a* path between the polygenic score and the ACE (*a* path) will also increase to $k^{*}\sqrt{\left(0.06\right)}$, where *k* reflects the ratio between the path from the polygenic score to the ACE, and the path from the polygenic score to internalising problems (*k* = *a* / *b*). Note that the SNP heritability estimate for childhood externalising problems that was used for this analysis is 9%^33^ (hence, *r* = 0.30). Supplementary Table 11 shows the method for estimating each of the original paths included in the correlation matrix.

To obtain a single estimate reflecting the proportion of the associations between ACEs and mental health outcomes captured by polygenic scores capturing SNP-based heritability, we averaged the results across 6 models for all ACEs (for internalising and externalising problems, separately). As described above for Hypothesis 2a, this was performed using the *MAd* package^125^.

We proposed to infer support for Hypothesis 2b (that a moderate proportion of the association is explained by polygenic scores) if polygenic scores capturing SNP-based heritability explained between 20% to 40% of the associations between ACEs and mental health outcomes on average. We planned to interpret alternative proportions of less than 5% as “very small”, proportions between 5% and 20% as “small”, and proportions of more than 40% as “large”.

### Sampling plan

#### Inclusion criteria and sample size

##### ALSPAC

We planned to include ALSPAC children if they had data on genotype that passed QC (see QC exclusions in Supplementary Table 8), ACEs (defined as responses to ≥ 50% of the questions in the assessments between birth and age 9 years for each ACE), internalising problems at age 10 (defined as responses to ≥ 50% of items assessing separation anxiety, social anxiety, general anxiety, and major depression on the Development and Wellbeing Assessment [DAWBA]) and externalising problems at age 10 (defined as responses to ≥ 50% of items assessing hyperkinesis/ADHD and conduct/oppositional disorders on the DAWBA). Based on a previous ALSPAC study using data on genotype and the DAWBA at age 10^126^, we expected the sample of complete cases to be N~5,900. However, to maximise sample size and reduce selection bias due to attrition, we proposed to use multiple imputation to impute missing values in the ACEs and internalising and externalising problems measures (see Supplementary Methods 2 for details of the inclusion criteria for imputation).

##### ABCD

We planned to include children from the ABCD Study if they had data on genotype that passed QC (see QC exclusions in Supplementary Table 8), ACEs (defined as responses to ≥ 50% to items assessing each ACE), internalising problems, and externalising problems at age 9/10 (defined as responses to ≥ 50% of relevant items on the CBCL). Based on previous ABCD studies using genotype data and ACEs/CBCL data, we expected the sample size to be between 4,700-5,400^42,127^. However, because we anticipated that the sample size may vary across different assessments (used to derive measures of ACEs and mental health), we proposed to use multiple imputation to maximise the sample size by imputing missing values in measures of ACEs and mental health (see Supplementary Methods 3 for details of the inclusion criteria for imputation).

##### Power calculations

We calculated power to test each of our hypotheses assuming a conservative minimum sample size of N=4,700, as the minimum expected sample sizes were 4,700 for the ABCD Study and 5,900 for ALSPAC. (Note that the ABCD Study was not originally included in the Stage 1 pre-registration, but we used it because the original dataset, the Child and Adolescent Twin Study in Sweden [CATSS], was not accessible after Stage 1 acceptance. The expected sample size for CATSS was 11,000). We conducted each power analysis using simulation (1,000 simulated datasets) in the *MASS*^128^ and *stats^112^* packages, and set the alpha level for statistical significance to 0.05. As described below, power to test each hypothesis was ≥0.95.

##### Hypothesis 1a

We calculated power to obtain a single effect size reflecting the average association between polygenic scores for mental health problems and ACEs across 48 logistic regression models (i.e., 8 polygenic scores x 6 ACEs). This analysis showed that power will be 0.96 to detect an average odds ratio of 1.04 for the effect of polygenic scores on ACEs using the ‘agg’ function in the *MAd* package^114^ (accounting for dependent effect sizes). An odds ratio of 1.04 is a conservative estimate as the average odds ratio for the associations between polygenic scores for mental health problems and ACEs in previous research^21–26^ was 1.10 (see https://osf.io/2uc4p/?view_only=2d9afc1b072b4507ba11ba8771aaab62 for details).

##### Hypothesis 1b

We calculated power to detect a significant difference in the associations between polygenic scores and ACEs according to the type of polygenic score, using a Wald test in *lavaan^117^*. This analysis showed that we will have 1.00 power to detect a difference across 8 effect sizes (reflecting the average effect of each polygenic score on ACEs), when the smallest and largest odds ratios differ by 0.11 (e.g., odds ratio=1.05 versus 1.16), with other effect sizes taking intermediate values. A simulation using a structural equation model (shown Supplementary Fig. 1) showed that these odds ratios are plausible assuming previously observed effects of polygenic scores on ACEs (odds ratios of between 1.03 and 1.16^21^), and average correlations of *r* = 0.06 between polygenic scores^23^ and *r* = 0.30 between ACEs in ALSPAC^77^.

##### Hypothesis 1c

Similarly to Hypothesis 1b, we calculated power to detect a significant difference in the associations between polygenic scores and ACEs according to the type of ACE, using a Wald test in *lavaan*^117^. This analysis showed that we will have 1.00 power to detect a difference across 6 effect sizes (reflecting the average effect of all polygenic scores on each ACE), when the smallest and largest odds ratios differ by 0.10 (e.g., odds ratio = 1.05 versus 1.15), with other effect sizes taking intermediate values. As described above, these effect sizes were found to be plausible in a simulation based on the structural equation model in Supplementary Fig. 1, assuming previously observed odds ratios for the effects of polygenic scores on different ACEs varying between 1.03 to 1.16^21^ and average correlations of *r* = 0.06 between polygenic scores^23^ and *r* = 0.30 between ACEs in ALSPAC^77^.

##### Hypothesis 2a

We calculated power for two analyses: (i) a structural equation model to estimate the proportion of the association between (individual) ACEs and mental health outcomes explained by polygenic scores, and (ii) an aggregate model to average the results across individual structural equation models. For the structural equation model (shown in Fig. 5C), power was 0.95 to detect the proportion of the association between ACEs and mental health explained by observed polygenic scores. This is assuming previously observed small independent effects of polygenic scores for mental health problems on ACEs (*r* = 0.03-0.07)^23^ and internalising and externalising problems (*r* = 0.01-0.05)^84^, small effects of individual ACEs on internalising and externalising problems (r=0.06)^129^, and average correlations between polygenic scores of *r* = 0.06^23^. For the aggregate model, power was 1.00 to detect an average proportion of 5% (of the association between ACEs and mental health explained by polygenic scores), assuming correlations of *r* = 0.30 between effect sizes. We consider 5% to be a conservative estimate of the likely proportion of the association between ACEs and mental health explained by multiple polygenic scores, given that prior studies have found that a single polygenic score can account for larger proportions of the associations between environmental exposures and mental health (e.g., 6%^30^ and 18%^28^).

##### Hypothesis 2b

We calculated power for a structural equation model with a single mediator (i.e., a polygenic score capturing additional genetic variance in the outcome), as shown in Fig. 5B. Power was 1.00 to detect the proportion of the association between ACEs and mental health explained by a polygenic score that captures SNP heritability in the outcome. This is assuming a path from the polygenic score to internalising problems of *r* = 0.24 (i.e., the square root of 0.06, as the SNP-based heritability of internalising problems is 6%^33^), a path from the polygenic score to the ACE of *r* = 0.07 (assuming that *k*=0.33, i.e., that the effect of the observed polygenic score on the ACE is a third of the size as the effect of the observed polygenic score on internalising problems), and a path from the ACE to internalising problems of *r* = 0.06 (as observed previously^129^). Note that power will be equally high for analyses on externalising problems because the SNP-based heritability of externalising problems is slightly higher than for internalising problems (9% versus 6%^33^). Furthermore, note that power will be ≥0.96 to aggregate these results to obtain an average proportion across models, assuming that the proportion will be 5% or greater (as tested above for Hypothesis 2a). This is because as the strength of the association between polygenic scores and mental health outcomes increases, the proportion of the association between ACEs and mental health explained by polygenic scores will increase^27^.

### Protocol registration

The Stage 1 protocol for this Registered Report was accepted in principle on 4 January 2021. The protocol, as accepted by the journal, can be found at https://doi.org/10.6084/m9.figshare.13580777.v1

## Supplementary Material

Supplement

## Figures and Tables

**Table 1 T1:** Design table summarising the study’s research questions, hypotheses, power calculations, analyses, and conditions for interpretation.

Question	Hypothesis	Sampling plan (e.g. power analysis)	Analysis plan	Interpretation given to different outcomes
Do children with genetic liability to mental health problems have an increased risk of ACEs?	1a) Polygenic scores for mental health problems will be associated with an increased risk of exposure to ACEs.	N=4,700 gives 0.96 power to detect an average odds ratio of 1.04 for the association between polygenic scores and ACEs using the ‘agg’ function in the MAd package^114^ (accounting for dependent effect sizes).	Logistic regression models testing the association between each polygenic score (including negative controls) and each ACE. Pool results from all logistic regression models in an aggregate meta-analysis model for associations between i) polygenic scores for mental health problems and ACEs, and ii) negative control polygenic scores and ACEs. Assess whether the 90% confidence interval (CI) for the pooled odds ratio for the association between polygenic scores for mental health problems and ACEs lies between 0.94 – 1.06 (equivalence bounds).	A positive and statistically significant pooled association between polygenic scores for mental health problems and ACEs will suggest that children with genetic liability to psychopathology have elevated risk of ACEs. A non-significant association will suggest absence of evidence for this. If CIs for this association are within the equivalence bounds, it will suggest that children with genetic liability to psychopathology do not have a meaningful increase in risk for ACEs. If the CIs do not fall within the equivalence bounds, it will suggest that the association is of meaningful magnitude. If the pooled association between negative control polygenic scores and ACEs is statistically significant, it will suggest that the results may be affected by biases in polygenic scores. If this association is non-significant, it will suggest that such biases do not affect the results. Hypothesis 1a will be supported if 1) the pooled association between polygenic scores for mental health problems and ACEs is statistically significant, 2) CIs for this association do not fall within the equivalence bounds, and 3) the pooled association between negative control polygenic scores and ACEs is non-significant.
Are polygenic scores for certain mental health problems more strongly associated with ACEs than other polygenic scores?	1b) Polygenic scores for different mental health problems equally predict exposure to ACEs.	N=4,700 gives 1.00 power to detect a significant difference of 0.11 in odds ratios reflecting the average association between different polygenic scores and ACEs, using a Wald test.	Structural equation model (SEM) to estimate the associations between each polygenic score and each ACE (Supplementary Fig. 1). Calculate the average association between each polygenic score with all ACEs. Wald test to assess whether the average association between each polygenic score with ACEs varies across polygenic scores. IF the Wald test is significant, conduct pairwise comparisons to assess which polygenic scores differ in prediction of ACEs Calculate differences in log odds ratios between average associations between different polygenic scores and ACEs, and assess whether the 90% CIs for the differences fall within -0.10 to 0.10 (equivalence bounds).	A statistically significant Wald test will suggest that polygenic scores differ in their association with ACEs. Follow-up pairwise comparisons will show which polygenic scores differ. A non-significant Wald test would suggest absence of evidence for differences between polygenic scores in association with ACEs. If the CIs for differences between polygenic scores in their associations with ACEs are within the equivalence bounds, it will suggest that there are not meaningful differences between polygenic scores in their association with ACEs. If the CIs do not fall within the equivalence bounds, it will suggest that differences are of meaningful magnitude. Hypothesis 1b will be supported if 1) the Wald test is non-significant, and 2) CIs for differences between polygenic scores are within the equivalence bounds.
Are some ACEs linked to greater polygenic risk for mental health problems than other ACEs?	1c) Parental mental illness, parental substance abuse, and parental criminality will be associated with higher polygenic risk for mental health problems relative to maltreatment, domestic violence, and parental separation.	N=4,700 gives 1.00 power to detect a significant difference of 0.10 in odds ratios reflecting the average association between polygenic scores and different ACEs, using a Wald test.	SEM to estimate the associations between each polygenic score and each ACE (Supplementary Fig. 1). Calculate the average association between each ACE and all polygenic scores. Wald test to assess whether the average effect of all polygenic scores on each ACE varies across ACEs. IF the Wald test is significant, conduct pairwise comparisons to assess which ACEs differ in the association with polygenic scores. Calculate differences in log odds ratios between average associations between different ACEs and polygenic scores, and assess whether the 90% CIs for the differences fall within -0.05-0.05 (equivalence bounds).	A statistically significant Wald test will suggest that ACEs differ in polygenic risk for mental health problems. Follow-up pairwise comparisons will show which ACEs differ. A non-significant Wald test would suggest absence of evidence for differences between ACEs in polygenic risk for mental health problems. If the CIs for differences between ACEs in their associations with polygenic scores are within the equivalence bounds, this will suggest that there are not meaningful differences between these ACEs in polygenic risk for mental health problems. If the CIs do not fall within the equivalence bounds, this will suggest that the differences are of meaningful magnitude. Hypothesis 1c will be supported if 1) the Wald test is significant, 2) pairwise comparisons show that parental mental illness, parental substance abuse, and parental criminality are associated with higher polygenic risk than other ACEs, and 3) confidence intervals for these differences are not within the equivalence bounds.
What proportion of the associations between ACEs and internalising and externalising problems are explained by observed polygenic scores for mental health problems?	2a) Observed polygenic scores will explain a small proportion (between 5% to 20%) of the associations between ACEs and internalising and externalising problems.	N=4,700 gives 0.95 power to detect the proportion of the association between ACEs and mental health explained by observed polygenic scores in a SEM. For the aggregate model, N=4,700 will give power of 1.00 to detect an average proportion of 5% (of the association between ACEs and mental health explained by polygenic scores).	SEMs (Fig. 5C) to test whether the associations between each ACE and each mental health outcome are mediated by polygenic scores (statistically equivalent to testing confounding). Calculate the proportion of the association between the ACE and mental health outcome explained by the polygenic scores. Pool results in an aggregate model to assess the average proportion of the associations between ACEs and mental health outcomes explained by observed polygenic scores. Repeat analyses using negative control polygenic scores.	The average proportion of associations between ACEs and mental health outcomes explained by observed polygenic scores will be interpreted as follows, broadly in line with guidance for interpreting effect sizes^123^: ○ <5% = “very small” ○ 5-20% = “small” ○ 20-40% = “moderate” ○ >40% = “large” Hypothesis 2a will be supported if 1) polygenic scores for mental health problems explain, on average, between 5% to 20% of the associations, and 2) the average proportion of the association explained by negative control polygenic scores is not significantly different from zero.
What proportion of the associations between ACEs and internalising and externalising problems are explained by polygenic scores which capture additional heritability in mental health problems?	2b) Polygenic scores that capture SNP heritability in internalising and externalising problems will explain a moderate proportion (between 20% to 40%) of the associations between ACEs and these outcomes.	N=4,700 gives 1.00 power to detect the proportion of the association between ACEs and mental health explained by increasingly powerful polygenic scores in a SEM.	SEM (Fig. 5B) to test whether the associations between each ACE and each mental health outcome are mediated by polygenic scores capturing SNP heritability in the outcome. Estimate model from a correlation matrix, modified to reflect additional genetic variance captured in the outcome^27,124^ and ACE according to the ratio observed based on the observed polygenic scores. Pool results in an aggregate model to assess the average proportion of the associations between ACEs and mental health outcomes explained by polygenic scores capturing SNP heritability.	The proportion of associations explained by polygenic scores capturing SNP-based heritability will be interpreted as specified above. Hypothesis 2b will be supported if polygenic scores capturing SNP-based heritability explain between 20% to 40% of the associations between ACEs and mental health outcomes on average.

Table legend: If findings differ between ALSPAC and the ABCD Study, we proposed to interpret this as reflecting: (1) differences between countries (the UK [ALSPAC] versus the USA [ABCD]), or (2) differences in historical time periods (as ALSPAC participants were born in 1991-1992 and ABCD participants were born in 2006-2008). Differences in results between cohorts are less likely to be due to polygenic scores (as the same GWAS summary statistics will be used for both cohorts), ACE measures (as both cohorts used similar questionnaires reported by parents and children), mental health measures (as both cohorts used similar parent-reported questionnaires) and timing of assessments (as ACEs were assessed between birth to age 9/10 in both cohorts, and mental health was assessed at age 10 in ALSPAC and age 9/10 in ABCD. Note that the ABCD Study was not originally included in the Stage 1 pre-registration, but we used it because the original replication cohort (CATSS) was not accessible after Stage 1 acceptance (detailed in “Methods – Change in replication cohort”).

## Data Availability

The ABCD Study anonymized data, including all assessment domains, are released annually to the research community. Information on how to access ABCD data through the NDA is available on the ABCD Study data-sharing webpage: https://abcdstudy.org/scientists_data_sharing.html. Instructions on how to create an NDA study are available at https://nda.nih.gov/training/modules/study.html. The ABCD data repository grows and changes over time. The ALSPAC data are not publicly available as informed consent for public data-sharing, and ethical approval for public data-sharing were not obtained from participants. Researchers can find details of how to apply for access to the ALSPAC dataset here: http://www.bristol.ac.uk/alspac/researchers/access/.

## Code availability

Analysis code can be found on https://github.com/jr-baldwin/ACEs_mental_health_RR.
