# Supplementary material for: A genetically informed Registered Report on adverse childhood experiences and mental health
Source: Nat Hum Behav. Author manuscript; Available in PMC 2023 Feb 25. (PMC7614239; doi:10.1038/s41562-022-01482-9)
Supplement: Supplement [file EMS159974-supplement-Supplement.docx]

**Supplementary Information**

[Supplementary Methods 1. The Child and Adolescent Twin Study in Sweden (CATSS). 3](#_Toc116464009)

[Supplementary Methods 2. Multiple imputation procedure for ALSPAC data 6](#_Toc116464010)

[Supplementary Methods 3. Multiple imputation procedure for ABCD Study data 6](#_Toc116464011)

[Supplementary Results 1. Complete case analysis 7](#_Toc116464012)

[Supplementary Results 2. Results using the pre-registered GWAS for bipolar disorder (Stahl et al., 2019). 10](#_Toc116464013)

[Supplementary Discussion. Issues with the genetic sensitivity analysis if the observed polygenic score for the outcome is more strongly associated with the exposure 13](#_Toc116464014)

[Supplementary Figure 1. Structural equation model to estimate the unique effects of polygenic scores for mental health problems on different ACEs. 14](#_Toc116464015)

[Supplementary Figure 2. Model to estimate the proportion of the associations between ACEs and mental health explained by negative control polygenic scores. 15](#_Toc116464016)

[Supplementary Figure 3. Associations between polygenic scores and ACEs in ALSPAC (complete cases). 16](#_Toc116464017)

[Supplementary Figure 4. Associations between polygenic scores and ACEs in ABCD (complete cases). 17](#_Toc116464018)

[Supplementary Figure 5. Pairwise differences between polygenic scores in their association with ACEs (complete cases). 18](#_Toc116464019)

[Supplementary Figure 6. Pairwise differences between ACEs in their association with polygenic risk for psychopathology (complete cases). 19](#_Toc116464020)

[Supplementary Table 1. Prevalence of ACEs in ALSPAC and the ABCD Study. 21](#_Toc116464021)

[Supplementary Table 2. Associations between ACEs and mental health after controlling for observed polygenic scores for mental health problems. 22](#_Toc116464022)

[Supplementary Table 3. Variance in ACEs and mental health problems explained by all polygenic scores for psychiatric disorders. 23](#_Toc116464023)

[Supplementary Table 4. Associations between ACEs and mental health after controlling for latent polygenic scores capturing additional heritability in mental health problems. 24](#_Toc116464024)

[Supplementary Table 5. Details of GWAS summary statistics used to derive polygenic scores. 25](#_Toc116464025)

[Supplementary Table 6. ACE definitions and measures in the ALSPAC cohort. 26](#_Toc116464026)

[Supplementary Table 7. Items from the Development and Wellbeing Assessment (DAWBA) used to derive mental health measures in ALSPAC. 27](#_Toc116464027)

[Supplementary Table 8. Genotyping and quality control procedures. 30](#_Toc116464028)

[Supplementary Table 9. ACE definitions and measures in the ABCD cohort. 31](#_Toc116464029)

[Supplementary Table 10. Items from the CBCL used to derive mental health measures in the ABCD Study. 32](#_Toc116464030)

[Supplementary Table 11. Method for estimating each original parameter included in the genetic sensitivity correlation matrix. 34](#_Toc116464031)

[Supplementary Table 12. Auxiliary variables included in the ALSPAC imputation model. 35](#_Toc116464032)

[Supplementary Table 13. Details of how variables were included in the ALSPAC imputation model. 37](#_Toc116464033)

[Supplementary Table 14. Details of variables included in the ABCD imputation model. 40](#_Toc116464034)

[Supplementary Table 15. ACE definitions and measures in the CATSS cohort. 41](#_Toc116464035)

[Supplementary Table 16. Associations between ACEs and mental health after controlling for observed polygenic scores for mental health problems (complete cases). 42](#_Toc116464036)

[Supplementary Table 17*.* Associations between ACEs and mental health after controlling for latent polygenic scores capturing additional heritability in mental health problems (complete cases). 43](#_Toc116464037)

[Supplementary References 44](#_Toc116464038)

# Supplementary Methods 1. The Child and Adolescent Twin Study in Sweden (CATSS).

***Ethics information***

CATSS was approved by the ethics committee at Karolinska Institutet and all participants gave informed consent.

***Design***

CATSS is a prospective longitudinal cohort study. A description of the sample and the measures is below.

***Sample***

The Child and Adolescent Twin Study in Sweden (CATSS) is a longitudinal study of twins born in Sweden since 1992. Since 2004, parents of twins have been invited to respond to a structured telephone interview about their children’s health and environment in connection with their 9^th^ birthday (cohort born July 1^st^ 1995 onward) or 12^th^ birthday (cohort born 1992 to 1995). The response rate is 67%^14^. Twins participating in the study are then assessed at ages 15, 18 and 24 years. Furthermore, the CATSS is linked with Swedish population-based registers, making it possible to obtain information about participants and their family members from national records as well as through the study assessments. Linkage is performed by identifying CATSS participants in the nationwide registers through their unique 10-digit civil registration numbers, which all Swedish residents are assigned. Family members of CATSS participants can also be identified through the Multi-Generation Register, which provides data on all individuals born in Sweden and their biological parents^15^. For further details on the CATSS sample, see Anckersäter et al.^14^.

***Measures***

*Adverse childhood experiences.* We proposed to assess six ACEs (maltreatment, domestic violence, parental mental illness, parental substance abuse, parental separation, and parental criminality) between birth and age 9 years, using two sources of information (Supplementary Table 15. First, we planned to use parent reports on the Life Stressor Checklist-Revised (LSC-R^16^) collected during a telephone interview when children were aged 9 years old. Second, we planned to use Swedish national registers (namely, the National Patient Register, the Prescribed Drug Register, the National Crime Register, The Register of Persons Suspected of Offenses, and the Total Population Register), where relevant, by linking CATSS participants via their 10-digit civil registration numbers. For each birth cohort of CATSS participants (e.g., from 1998 to 2010), we proposed to search in the Swedish national registers for ACEs experienced prior to their 9^th^ birthday (e.g., for a child born in 1998, to search from 1998-2007, or for a child born in 2010, to search from 2010-2019). We proposed to derive binary measures reflecting exposure to each ACE according to definitions and criteria for exposure shown in Supplementary Table 15.

*Mental health problems.* To maximise comparability with outcomes assessed in ALSPAC, we planned to focus on internalising problems and externalising problems at age 9 assessed through parent reports via a structured telephone interview. We proposed to assess *internalising problems* through the Screen for Child Anxiety Related Emotional Disorders (SCARED)^17^ and the Short Mood and Feelings Questionnaire (SMFQ)^18^. The SCARED is a 41-item measure assessing anxiety (including panic/somatic problems, generalized anxiety, separation anxiety, social phobia, and school phobia), and the SMFQ is a 13-item measure assessing depression (including low mood, anhedonia, tiredness, restlessness, low self-worth, crying, cognitive difficulties and loneliness). Both measures have shown good reliability and validity (for SCARED, see^17,19-21^; for SMFQ, see^22-24^). The scale for the SCARED ranges from 0-82 and the scale for the SMFQ ranges from 0-26^25^. To derive one overall measure of internalising problems, we proposed to first calculate the mean for each of the two measures (SCARED, SMFQ) for participants with data for ≥50% of the items, and then standardise both scores and then sum them, before standardising the overall single measure.

We proposed to assess *externalising problems* through the Autism-Tics, ADHD, and other Comorbidities Inventory (A-TAC), which is a 96-item measure assessing child neurodevelopmental and psychiatric disorders with good to excellent reliability and validity^26-29^. We planned to use the Opposition module (5 items, scale from 0-5), the Conduct module (5 items, scale from 0-5), the Concentration and Attention module (9 items, scale from 0-9), and the Impulsiveness and Activity module (10 items, scale from 0-10), to assess externalising problems. To derive one overall measure of externalising problems, we proposed to first calculate the mean for each of the four modules (Opposition, Conduct, Concentration and Attention, and Impulsiveness and Activity) for participants with data for ≥50% of the items, and then standardise all four scores and then sum them, before standardising the overall single measure.

*Genotyping and quality control.* CATSS twins have been genotyped using the Illumina Infinium PsychArray-24 BeadChip and the Illuminia Infinium Global Screening Array-24 BeadChip, carried out by SNP&SEQ Technologies in Uppsala, Sweden. We planned to carry out quality control in PLINK^30^, implementing the CATSS protocol for genetic data^31^ which adheres to standard guidelines^32^. Further details are provided in Supplementary Table 8.

*Polygenic scores for mental health problems.* We proposed to derive polygenic scores for mental health problems and negative controls using the same procedure as described above for ALSPAC participants, and residualizing polygenic scores for genotyping batch as CATSS participants have been genotyped in two waves.

**Sampling plan for CATSS**

*Inclusion criteria and sample size*

*CATSS*. We planned to include CATSS participants if they were born in 1998 or later (as participants born earlier were not assessed for ACEs), and had complete data on genotype, six ACEs assessed at age 9 (defined as responses to all relevant assessment items on the Life Stressor Checklist-Revised), internalising problems at age 9 (defined as responses to ≥ 50% of assessment items on the Screen for Child Anxiety Related Emotional Disorders and the Short Mood and Feelings Questionnaire) and externalising problems at age 9 (defined as responses to ≥ 50% of items on the Opposition, Conduct, Concentration and Attention, and Impulsiveness and Activity subscales of the Autism-Tics, ADHD, and other Comorbidities Inventory). The CATSS data management team (who are not involved in this study) estimated that this will comprise 11,144 participants.

# Supplementary Methods 2. Multiple imputation procedure for ALSPAC data

Previous analyses on the ALSPAC cohort have suggested that participants with complete data for adverse childhood experiences (ACEs) are more affluent than the full cohort^33^, and thus including only these participants will lead to lower ACE prevalence estimates and may induce selection bias. To address this, we used multiple imputation to replace missing values in the measures of ACEs and mental health (assessed through the DAWBA).

*Inclusion criteria for imputation.* We imputed data for participants with genotype data, at least 10% of questionnaire responses used to define exposure to ACEs between birth and age 9 (as in previous ALSPAC studies^33,34^), and at least one DAWBA measure assessed at ages 7, 10, or 13 years. The complete case sample comprised participants with genotype data, ≥ 50% of the questionnaire responses for each ACE, and DAWBA data at age 10.

*Multiple imputation procedure*. We performed multiple imputation using the *Amelia* package^35^ in R, and created 50 imputed datasets. As recommended by guidelines on using the ACE measures, we included auxiliary variables in the imputation model that are associated with missingness, ACEs, and DAWBA measures (Supplementary Table 12). A description of how each variable was handled in the imputation model is provided in Supplementary Table 13. The 50 imputed datasets were then analysed using the methods described in the ‘Statistical analysis’ section. Estimates were pooled across imputed datasets using Rubin’s rules. We conducted primary analyses on the imputed sample, but report findings from the complete case sample in the Supplementary Results for transparency.

# Supplementary Methods 3. Multiple imputation procedure for ABCD Study data

Inclusion criteria for imputation. We imputed data for participants with genotype data and at least 10% of questionnaire responses used to define exposure to ACEs between birth and age 9/10, similar to the ALSPAC Study. The complete case sample comprised participants with data for genotype, ACEs, and mental health at age 9/10.

*Multiple imputation procedure*. We used the same procedure to impute missing data in the ABCD Study as in ALSPAC. Supplementary Table 14 provides a list of these variables and a description of how each variable was handled in the imputation model.

# Supplementary Results 1. Complete case analysis

We repeated the analysis in the samples of complete cases (N=4,106 in ALSPAC and N=4,662 in the ABCD Study).

**1a) Do children with genetic liability to mental health problems have an increased risk of ACEs?**

*ALSPAC*. Children with greater genetic liability to psychopathology had a small increase in odds of ACEs (pooled OR=1.05, 95% CI=1.00-1.10; Supplementary Figure 2A) but this was not statistically significant (p=0.057). The 90% CIs for this pooled association (1.01-1.09) did not fall within the equivalence bounds (0.94-1.06). Negative control polygenic scores were not associated with ACEs (pooled OR=0.97, 95% CI=0.92-1.02, p=0.25; Supplementary Figure 2B).

*ABCD.* Children in the ABCD cohort with greater genetic liability to psychopathology had a higher odds of ACEs (pooled OR=1.09, 95% CI=1.03-1.15, p=0.0045, Supplementary Figure 3A), and the 90% CIs (1.04-1.14) did not fall within the equivalence bounds (0.94-1.06). Conversely, negative control polygenic scores were not associated with ACEs (pooled OR=1.02, 95% CI=0.96-1.07, p=0.62; Supplementary Figure 3B).

**1b) Are polygenic scores for certain mental health problems more strongly associated with ACEs than other polygenic scores?**

*ALSPAC*. A non-significant Wald test (p=0.079) suggested an absence of evidence for differences between polygenic scores in association with ACEs. Equivalence tests found that the majority of polygenic scores were equally associated with ACEs, with the exception of differences between depression with all other polygenic scores (Supplementary Figure 4A).

*ABCD*. Polygenic scores for various mental health problems showed different associations with ACEs in the ABCD Study (Wald-test p-value=2.21x10^-9^). Pairwise comparisons showed stronger effects of the polygenic scores for ADHD, antisocial behaviour, bipolar disorder, depression, and schizophrenia than other polygenic scores (particularly for alcohol dependence and autism; see Supplementary Figure 4B for specific comparisons). The 90% CIs for these differences did not fall within the equivalence bounds.

**1c) Are some ACEs linked to greater polygenic risk for mental health problems than other ACEs?**

*ALSPAC*. In the ALSPAC Study, there was no evidence for differences between ACEs in polygenic risk for mental health problems (Wald-test p-value=0.17). Furthermore, equivalence tests suggested that the majority of ACEs were associated with equal polygenic risk of mental health problems, as the 90% CIs for the differences between ACEs fell inside the equivalence bounds (-0.05 to 0.05 on the log odds ratio; Supplementary Figure 5A).

*ABCD*. Similar to ALSPAC, in the ABCD cohort, differences between ACEs in polygenic risk for mental health problems were not found (Wald-test p-value=0.10). Equivalence tests also suggested that the majority of ACEs were associated with equal polygenic risk of mental health problems, as the 90% CIs for the differences between ACEs fell inside the equivalence bounds (-0.05 to 0.05 on the log odds ratio; Supplementary Figure 5B).

**2a) What proportion of the associations between ACEs and internalising and externalising problems is explained by observed polygenic scores for mental health problems?**

*ALSPAC*. On average, polygenic scores for mental health problems explained a very small average proportion of the associations between ACEs with internalising problems (4.1%, 95% CI=0.3-7.8%) and externalising problems (4.5%, 95% CI=0.2-8.9%). Results for specific associations between ACEs with internalising and externalising problems are shown in Supplementary Table 16A. In contrast, negative control polygenic scores did not explain any of the associations between ACEs with internalising problems (-0.2%, 95% CI=-0.9;0.5%, p=0.58) or externalising problems (-0.2%, 95% CI=-0.9;0.5%, p=0.55).

*ABCD*. Similar to ALSPAC, in the ABCD cohort, polygenic scores explained a very small average proportion of the associations between ACEs with internalising problems (3.3%, 95% CI=1.2-5.5%) and a small average proportion of the associations with externalising problems (5.4%, 95% CI=3.5-7.3%, with specific associations shown in Supplementary Table 16B). Conversely, negative control polygenic scores did not explain any of the associations between ACEs with internalising problems (0.1%, 95% CI=-0.4;0.6%, p=0.74) or externalising problems (0.1%, 95% CI=-0.3;0.5%, p=0.69).

**2b) What proportion of the associations between ACEs and internalising and externalising problems is explained by polygenic scores which capture additional heritability in mental health problems?**

*ALSPAC.* On average, a latent polygenic score capturing SNP heritability in internalising problems explained a large proportion of the associations between ACEs and internalising problems in ALSPAC (91.6%; 95% CI=78.9-100%), with proportions ranging from 56.7% for parental mental illness to 100% for domestic violence, parental substance abuse, criminality and separation (Supplementary Table 17A). The associations between ACEs and externalising problems were also largely explained by a latent polygenic score capturing SNP heritability in externalising problems (average proportion=86.7%, 95% CI=73.9-99.5%, ranging from 68.1% [maltreatment] to 100% [parental substance abuse, criminality, and separation]; Supplementary Table 17A).

*ABCD.* Similar to ALSPAC, in the ABCD cohort, a latent polygenic score capturing SNP heritability in internalising problems explained a large average proportion of the associations between ACEs and internalising problems (70.6%; 95% CI=56.0-85.1%), with proportions ranging from 22.5% for parental psychopathology to 100% for parental criminality and separation (Supplementary Table 17B). The associations between ACEs and externalising problems were also largely explained by a latent polygenic score capturing SNP heritability in externalising problems (average proportion=63.9%, 95 CI=45.8-81.9%, ranging from 28.6% [maltreatment] to 100% [parental criminality]; Supplementary Table 17B).

# Supplementary Results 2. Results using the pre-registered GWAS for bipolar disorder (Stahl et al., 2019).

We repeated the main analyses using a polygenic score for bipolar disorder derived from the originally proposed GWAS from Stahl and colleagues (2019)^9^ rather than the updated GWAS from Mullins and colleagues (2021)^3^.

**1a) Do children with genetic liability to mental health problems have an increased risk of ACEs?**

*ALSPAC*. Children with greater genetic liability to psychopathology had a small increase in odds of ACEs (pooled OR=1.05, 95% CI=1.01-1.10, p=0.0089). The 90% CIs for this pooled association (1.02-1.09) did not fall within the equivalence bounds (0.94-1.06).

*ABCD.* Children in the ABCD cohort with greater genetic liability to psychopathology had a higher odds of ACEs (pooled OR=1.08, 95% CI=1.03-1.14, p=0.0033), and the 90% CIs (1.04-1.13) did not fall within the equivalence bounds (0.94-1.06).

**1b) Are polygenic scores for certain mental health problems more strongly associated with ACEs than other polygenic scores?**

*ALSPAC*. Polygenic scores for various mental health problems were differentially associated with ACEs (Wald-test p-value=0.009). Pairwise comparisons showed that the polygenic scores for depression, ADHD and schizophrenia predicted ACEs more strongly than various other polygenic scores (particularly for autism and alcohol dependence; Supplementary Figure 6A). The polygenic score for depression also predicted ACEs more strongly than the bipolar disorder and antisocial behaviour polygenic scores. The 90% CIs for these differences did not fall within the pre-specified equivalence bounds (-0.10 to 0.10 on the log odds scale; Figure 6A), suggesting that the differences were of a meaningful size.

*ABCD*. Polygenic scores for various mental health problems showed different associations with ACEs in the ABCD Study (Wald-test p-value=2.53x10^-9^). Pairwise comparisons showed stronger effects of the polygenic scores for ADHD, antisocial behaviour, bipolar disorder, depression, and schizophrenia than other polygenic scores (particularly for alcohol dependence and autism; see Supplementary Figure 6B for specific comparisons). The 90% CIs for these differences did not fall within the equivalence bounds.

**1c) Are some ACEs linked to greater polygenic risk for mental health problems than other ACEs?**

*ALSPAC*. In the ALSPAC Study, there was no evidence for differences between ACEs in polygenic risk for mental health problems (Wald-test p-value=0.33).

*ABCD*. Similar to ALSPAC, in the ABCD cohort, differences between ACEs in polygenic risk for mental health problems were not found (Wald-test p-value=0.08).

**2a) What proportion of the associations between ACEs and internalising and externalising problems is explained by observed polygenic scores for mental health problems?**

*ALSPAC*. On average, polygenic scores for mental health problems explained a very small average proportion of the associations between ACEs with internalising problems (4.3%, 95% CI=1.9-6.8%) and a small proportion for externalising problems (5.9%, 95% CI=2.9-8.9%).

*ABCD*. In the ABCD cohort, polygenic scores explained a very small average proportion of the associations between ACEs with internalising problems (3.1%, 95% CI=1.1-5.0%) and a small average proportion of the associations with externalising problems (5.1%, 95% CI=3.4-6.9%).

**2b) What proportion of the associations between ACEs and internalising and externalising problems is explained by polygenic scores which capture additional heritability in mental health problems?**

*ALSPAC.* On average, a latent polygenic score capturing SNP heritability in internalising problems explained a large proportion of the associations between ACEs and internalising problems in ALSPAC (90.7%; 95% CI=80.3-100%). The associations between ACEs and externalising problems were also largely explained by a latent polygenic score capturing SNP heritability in externalising problems (average proportion=77.0%, 95 CI=59.8-94.1%).

*ABCD.* Similarly to ALSPAC, in the ABCD cohort, a latent polygenic score capturing SNP heritability in internalising problems explained a large average proportion of the associations between ACEs and internalising problems (68.2%; 95% CI=55.3-81.1%). The associations between ACEs and externalising problems were also largely explained by a latent polygenic score capturing SNP heritability in externalising problems (average proportion=60.3%, 95 CI=53.6-67.0%).

# Supplementary Discussion. Issues with the genetic sensitivity analysis if the observed polygenic score for the outcome is more strongly associated with the exposure

The genetic sensitivity analysis aims to estimate genetic confounding under a scenario in which the polygenic score captures SNP heritability in the outcome. As described in the Methods, this involves estimating a structural equation model (Extended Data Fig. 2A) from a correlation matrix. This matrix includes correlations between the polygenic score and the ACE (*a* path), the polygenic score and the mental health outcome (*b* path), and the ACE and the mental health outcome (*cp* path). This correlation matrix is used to estimate a model where a latent factor captures additional genetic variance in outcome. For illustration purposes, this is equivalent to modifying the correlation matrix to reflect the additional SNP-based heritability. For example, if the SNP-based heritability of parent-reported childhood internalising problems is 6%^13^, the correlation coefficient from the polygenic score to internalising problems (*b* path) will be changed to *r* = 0.24 (calculated by taking the square-root of 0.06). The correlation coefficient for the path between the polygenic score and the ACE (*a* path) will also increase to *k**√(0.06), where *k* reflects the ratio between the path from the polygenic score to the ACE, and the path from the polygenic score to internalising problems (*k* = *a*/ *b*). Importantly, if the correlation between the observed polygenic score and the ACE (*a* path) is greater than the correlation between the observed polygenic score and the mental health outcome (*b* path), the *k* ratio will be greater than 1. For example, in ALSPAC, the correlation between polygenic scores for psychiatric disorders and internalising problems is 0.066, and 0.134 for parental mental illness, making *k* = 2.03. This is likely because the polygenic scores that we used were derived from adult psychiatric disorders and may therefore better predict some ACEs than the child outcome. As a result, under a SNP heritability scenario, the correlation coefficient for the path between the polygenic score and the ACE (*a* path) will increase to a value that is larger than the SNP heritability of the outcome (e.g., the *a* path from the polygenic score to parental mental illness would increase from *r* = 0.134 to *r* = 0.50, given 2.03*√(0.06)). This approximates a scenario whereby a polygenic score for the outcome captures more heritability in the exposure than the full SNP heritability of the outcome, which seems conceptually unlikely. As a result, the genetic confounding effect may be over-estimated given the implausibly large correlation between the polygenic score and exposure. This can be understood as a mismatch between what the observed polygenic score captures (i.e. as much of the exposure as the outcome) and what the heritability correction is based on (i.e. the SNP-heritability of the outcome). Therefore, as recommended in the manuscript, these analyses would benefit from using well powered GWAS that reflect as closely as possible the measured outcomes, which would, in all likelihood, lead to a substantially higher polygenic score-outcome association compared to the polygenic score-exposure association.

Supplementary Figure 1. Structural equation model to estimate the unique effects of polygenic scores for mental health problems on different ACEs.

Note: The top row of boxes (“PGS_1” to “PGS_8”) represent 8 polygenic scores for mental health problems, and the bottom row of boxes (“ACE_1” to “ACE_6”) represent 6 adverse childhood experiences. Solid lines represent estimated parameters, double-headed arrows represent correlations, single-headed arrows represent coefficients, and triangles above or below variables represent variances

Supplementary Figure 2. Model to estimate the proportion of the associations between ACEs and mental health explained by negative control polygenic scores.

Note. ACE represents the adverse childhood experience, MH represents the mental health outcome (e.g., internalising problems or externalising problems) and PGS_1 and PGS_2 represent the negative control polygenic scores (indexing genetic liability to left handedness and cataracts, respectively). Solid lines represent estimated parameters, double-headed arrows represent correlations, and single-headed arrows represent coefficients.

Supplementary Figure 3. Associations between polygenic scores and ACEs in ALSPAC (complete cases).

Note. Data are presented as odds ratios +/- 95% CIs, obtained from logistic regression models. Panel A shows associations between polygenic scores for mental health problems and ACEs, Panel B shows associations between negative control polygenic scores and ACEs. P-values for individual associations between polygenic scores and ACEs are from two-sided tests and are FDR corrected. N=4,106 participants.

Supplementary Figure 4. Associations between polygenic scores and ACEs in ABCD (complete cases).

Note. Data are presented as odds ratios +/- 95% CIs, obtained from logistic regression models. Panel A shows associations between polygenic scores for mental health problems and ACEs, Panel B shows associations between negative control polygenic scores and ACEs. P-values for individual associations between polygenic scores and ACEs are from two-sided tests and are FDR corrected. N=4,662 participants.

# Supplementary Figure 5. Pairwise differences between polygenic scores in their association with ACEs (complete cases).

Note: Data are presented as log odds differences +/- 90% CIs. Red dashed lines show the equivalence bounds. P-values (two-sided tests) are for the difference in log odds ratio between polygenic scores. N=4,106 independent participants in ALSPAC (Panel A) and N=4,662 independent participants in ABCD (Panel B).

# Supplementary Figure 6. Pairwise differences between ACEs in their association with polygenic risk for psychopathology (complete cases).

**A. ALSPAC**

**B. ABCD**

Note: Data are presented as log odds differences +/- 90% CIs. Positive effect sizes reflect the first labelled ACE having a stronger positive average association with polygenic risk for psychopathology; negative effect sizes reflect the second labelled ACE having a stronger positive association with polygenic risk for psychopathology. The red dashed lines show the equivalence bounds. N=4,106 independent participants in ALSPAC (Panel A) and N=4,662 independent participants in ABCD (Panel B).

**Supplementary Figure 7**. Pairwise differences between polygenic scores in their association with ACEs, using the Stahl GWAS for bipolar disorder

Note: Data are presented as log odds differences +/- 90% CIs. Red dashed lines show the equivalence bounds. P-values (two-sided tests) are for the difference in log odds ratio between polygenic scores. N=6,411 independent participants in ALSPAC (Panel A) and N=4,996 independent participants in ABCD (Panel B).

| **ACE** | **Imputed sample** | **Complete case sample** |
| --- | --- | --- |
| **ALSPAC** |  |  |
| Maltreatment | 24.24 | 19.09 |
| Domestic violence | 21.05 | 17.54 |
| Parental mental illness | 38.41 | 35.46 |
| Parental substance abuse | 10.72 | 8.33 |
| Parental criminality | 6.95 | 6.04 |
| Parental separation | 20.75 | 14.00 |
| **ABCD** |  |  |
| Maltreatment | 4.89 | 4.63 |
| Domestic violence | 30.86 | 30.61 |
| Parental mental illness | 40.56 | 40.48 |
| Parental substance abuse | 18.71 | 18.21 |
| Parental criminality | 2.98 | 2.68 |
| Parental separation | 22.20 | 21.51 |

# Supplementary Table 1. Prevalence of ACEs in ALSPAC and the ABCD Study.

The sample sizes of imputed data are n=6,411 for ALSPAC and n=4,996 for ABCD. The complete case sample sizes are n=4,106 in ALSPAC and n=4,662 in ABCD. We do not present descriptive statistics for polygenic scores, internalising problems or externalising problems because these variables were standardised to mean=0, standard deviation=1.

# Supplementary Table 2. Associations between ACEs and mental health after controlling for observed polygenic scores for mental health problems.

|  | 1. **ALSPAC** | | | | 1. **ABCD** | | | |
| --- | --- | --- | --- | --- | --- | --- | --- | --- |
|  | **Total association** | **Adjusted association** | **Genetic confounding** | **Prop. genetically confounded** | **Total association** | **Adjusted association** | **Genetic confounding** | **Prop. genetically confounded** |
| **Internalising problems** | | | | | | | | |
| Maltreatment | 0.110 (0.086-0.135) | 0.106 (0.082-0.131) | 0.004 (0.002-0.006) | 0.037 (0.014-0.059) | 0.121 (0.084-0.159) | 0.120 (0.082-0.158) | 0.002 (-0.001-0.004) | 0.013 (-0.008-0.034) |
| Domestic violence | 0.058 (0.033-0.084) | 0.055 (0.029-0.080) | 0.004 (0.001-0.006) | 0.065 (0.024-0.106) | 0.094 (0.067-0.121) | 0.091 (0.064-0.118) | 0.003 (0.000-0.006) | 0.029 (-0.003-0.061) |
| Parental mental illness | 0.177 (0.153-0.200) | 0.172 (0.149-0.196) | 0.005 (0.002-0.008) | 0.027 (0.011-0.044) | 0.281 (0.257-0.304) | 0.277 (0.254-0.301) | 0.004 (0.000-0.008) | 0.014 (0.001-0.027) |
| Parental substance abuse | 0.043 (0.017-0.068) | 0.039 (0.014-0.064) | 0.003 (0.001-0.006) | 0.087 (0.021-0.153) | 0.146 (0.119-0.173) | 0.141 (0.115-0.168) | 0.005 (0.001-0.009) | 0.031 (0.004-0.059) |
| Parental criminality | 0.046 (0.020-0.071) | 0.046 (0.021-0.071) | -0.001 (-0.002-0.001) | -0.012 (-0.055-0.031)^X^ | 0.080 (0.048-0.113) | 0.077 (0.044-0.110) | 0.003 (0.000-0.006) | 0.042 (0.003-0.081) |
| Parental separation | 0.056 (0.030-0.081) | 0.053 (0.027-0.078) | 0.003 (0.001-0.006) | 0.058 (0.017-0.099) | 0.100 (0.073-0.127) | 0.095 (0.068-0.123) | 0.005 (0.001-0.009) | 0.049 (0.004- 0.094) |
| **Externalising problems** | | | | | | | | |
| Maltreatment | 0.144 (0.120-0.168) | 0.139 (0.115-0.163) | 0.005 (0.002-0.009) | 0.037 (0.013-0.062) | 0.157 (0.118-0.196) | 0.153 (0.114-0.193) | 0.004 (0.001-0.008) | 0.026 (0.004-0.049) |
| Domestic violence | 0.088 (0.063-0.113) | 0.082 (0.057-0.107) | 0.007 (0.003-0.010) | 0.075 (0.032-0.117) | 0.162 (0.136-0.188) | 0.157 (0.130-0.183) | 0.006 (0.002-0.009) | 0.035 (0.013-0.057) |
| Parental mental illness | 0.157 (0.133-0.181) | 0.151 (0.127-0.175) | 0.006 (0.002-0.010) | 0.039 (0.014-0.063) | 0.272 (0.248-0.296) | 0.264 (0.240-0.288) | 0.009 (0.005-0.014) | 0.033 (0.018-0.048) |
| Parental substance abuse | 0.069 (0.049-0.090) | 0.062 (0.041-0.083) | 0.008 (0.005-0.010) | 0.109 (0.068-0.150) | 0.188 (0.162-0.214) | 0.178 (0.152-0.204) | 0.011 (0.006-0.015) | 0.056 (0.032-0.081) |
| Parental criminality | 0.056 (0.030-0.081) | 0.057 (0.032-0.082) | -0.001 (-0.004-0.002) | -0.021 (-0.083-0.040)^X^ | 0.117 (0.081-0.153) | 0.110 (0.073-0.146) | 0.008 (0.004-0.011) | 0.066 (0.031-0.101) |
| Parental separation | 0.091 (0.066-0.116) | 0.081 (0.056-0.106) | 0.010 (0.006-0.013) | 0.109 (0.069-0.149) | 0.144 (0.117-0.171) | 0.132 (0.105-0.159) | 0.013 (0.008-0.017) | 0.087 (0.051-0.122) |

Note. Prop=proportion. Results are presented as standardized coefficients, converted from raw Cohen’s d values for comparability with findings from the genetic sensitivity analysis (used to test Hypothesis 2b).

^X^ In ALSPAC, the proportion of the associations between parental criminality with internalising and externalising problems explained by polygenic scores was less than 0. This was because the proportion genetically confounded was calculated from a mediation model (see Figure 7**)** and the direct and indirect effects were in opposite directions.

# Supplementary Table 3. Variance in ACEs and mental health problems explained by all polygenic scores for psychiatric disorders.

|  | **ALSPAC** | | **ABCD** | |
| --- | --- | --- | --- | --- |
| **Outcome** | ***r*^2^** | ***r*** | ***r*^2^** | ***r*** |
| **ACEs** |  |  |  |  |
| Maltreatment | 0.011 | 0.106 | 0.009 | 0.095 |
| Domestic violence | 0.009 | 0.095 | 0.006 | 0.076 |
| Parental psychopathology | 0.018 | 0.134 | 0.033 | 0.182 |
| Parental substance abuse | 0.014 | 0.118 | 0.025 | 0.157 |
| Parental criminality | 0.004 | 0.061 | 0.035 | 0.187 |
| Parental separation | 0.015 | 0.122 | 0.037 | 0.193 |
| **Mental health** |  |  |  |  |
| Internalising problems | 0.004 | 0.066 | 0.007 | 0.085 |
| Externalising problems | 0.015 | 0.124 | 0.016 | 0.125 |

Note. Correlations were estimated by taking the square root of the r^2^ value from a structural equation model including all polygenic scores for psychiatric disorders and either ACEs or internalising/externalising problems as the outcome.

# Supplementary Table 4. Associations between ACEs and mental health after controlling for latent polygenic scores capturing additional heritability in mental health problems.

Note. Prop=proportion. Results are presented as standardized coefficients.

^X^Confidence intervals could not be reliably computed and therefore these estimates should be interpreted with caution.

|  | 1. **ALSPAC** | | | | 1. **ABCD** | | | |
| --- | --- | --- | --- | --- | --- | --- | --- | --- |
|  | **Total association** | **Adjusted association** | **Genetic confounding** | **Prop. genetically confounded** | **Total association** | **Adjusted association** | **Genetic confounding** | **Prop. genetically confounded** |
| **Internalising problems** | | | | | | | | |
| Maltreatment | 0.111 (0.086-0.135) | 0.017 (-0.038-0.072) | 0.094 (0.049-0.139) | 0.846 (0.371-1.320) | 0.121 (0.094-0.149) | 0.059 (0.020-0.099) | 0.062 (0.036-0.088) | 0.512 (0.260-0.764) |
| Domestic violence | 0.069 (0.051-0.087) | 0.000 (0.000-0.000) ^X^ | 0.069 (0.051-0.087) | 1.000 (1.000-1.000) ^X^ | 0.094 (0.066-0.121) | 0.043 (0.005-0.080) | 0.051 (0.027-0.075) | 0.544 (0.236-0.853) |
| Parental mental illness | 0.180 (0.156-0.204) | 0.078 (0.022-0.133) | 0.102 (0.059-0.146) | 0.569 (0.294-0.845) | 0.292 (0.265-0.318) | 0.228 (0.193-0.263) | 0.064 (0.053-0.075) | 0.220 (0.167-0.272) |
| Parental substance abuse | 0.070 (0.053-0.087) | 0.000 (0.000-0.000) ^X^ | 0.070 (0.053-0.087) | 1.000 (1.000-1.000) ^X^ | 0.144 (0.117-0.172) | 0.023(-0.047-0.093) | 0.121 (0.066-0.177) | 0.840 (0.376-1.303) |
| Parental criminality | 0.050 (0.031-0.068) | 0.000 (0.000-0.000) ^X^ | 0.050 (0.031-0.068) | 1.000 (1.000-1.000) ^X^ | 0.101 (0.082-0.120) | 0.000 (NA-NA)^X^ | 0.101 (0.082-0.120) | 1.000 (1.000-1.000)^X^ |
| Parental separation | 0.077 (0.059-0.094) | 0.000 (0.000-0.000) ^X^ | 0.077 (0.059-0.094) | 1.000 (1.000-1.000) ^X^ | 0.112 (0.092-0.132) | 0.000 (NA-NA)^X^ | 0.112 (0.092-0.132) | 1.000 (1.000-1.000)^X^ |
| **Externalising problems** | | | | | | | | |
| Maltreatment | 0.146 (0.121-0.170) | 0.074 (0.041-0.106) | 0.072 (0.052-0.092) | 0.494 (0.328-0.661) | 0.159 (0.131-0.186) | 0.095 (0.060-0.131) | 0.063 (0.043-0.084) | 0.399 (0.248-0.550) |
| Domestic violence | 0.088 (0.063-0.112) | 0.020 (-0.013-0.053) | 0.068 (0.046-0.090) | 0.769 (0.438-1.101) | 0.163 (0.135-0.190) | 0.112 (0.079-0.145) | 0.051 (0.031-0.070) | 0.312 (0.183-0.441) |
| Parental mental illness | 0.159 (0.135-0.183) | 0.069 (0.033-0.105) | 0.090 (0.067-0.113) | 0.564 (0.385-0.743) | 0.282 (0.255-0.309) | 0.197 (0.161-0.233) | 0.085 (0.068-0.102) | 0.302 (0.226-0.377) |
| Parental substance abuse | 0.071 (0.055-0.086) | 0.000 (0.000-0.000) ^X^ | 0.071 (0.055-0.086) | 1.000 (1.000-1.000) ^X^ | 0.187 (0.160-0.215) | 0.070 (0.021-0.118) | 0.118 (0.085-0.150) | 0.628 (0.407-0.849) |
| Parental criminality | 0.055 (0.031-0.080) | 0.011 (-0.019-0.042) | 0.044 (0.024-0.063) | 0.794 (0.310-1.279) | 0.123 (0.103-0.142) | 0.000 (0.000-0.000)^X^ | 0.123 (0.103-0.142) | 1.000 (1.000-1.000)^X^ |
| Parental separation | 0.091 (0.066-0.115) | 0.003 (-0.035-0.040) | 0.088 (0.062-0.114) | 0.970 (0.566-1.374) | 0.141 (0.113-0.169) | 0.003(-0.056-0.062) | 0.138 (0.094-0.183) | 0.978 (0.566-1.391) |

# Supplementary Table 5. Details of GWAS summary statistics used to derive polygenic scores.

| **Domain, trait** | **Total N** | **N cases** | **N controls** | **SNP h^2^** | **Year published** | **Reference** |
| --- | --- | --- | --- | --- | --- | --- |
| **Mental health** |  |  |  |  |  |  |
| Major depressive disorder | 500,199 | 170,756 | 329,443 | 0.089 | 2019 | 1 |
| Anxiety disorder | 83,566 | 25,453 | 58,113 | 0.260 | 2019 | 2 |
| Bipolar disorder | 413,466 | 41,917 | 371,549 | 0.186 | 2021 | 3 |
| Autism | 46,350 | 18,381 | 27,969 | 0.118 | 2019 | 4 |
| ADHD | 53,293 | 19,099 | 34,194 | 0.216 | 2019 | 5 |
| Antisocial behaviour^1,2^ | 83,674 | - | - | 0.084 | 2021 | 6 |
| Alcohol use disorders^1^ | 121,604 | - | - | 0.120 | 2019 | 7 |
| Schizophrenia | 105,318 | 40,675 | 64,643 | 0.200 | 2018 | 8 |
| *GWASs included in Stage 1 protocol^3^* |  |  |  |  |  |  |
| Bipolar disorder | 51,710 | 20,352 | 31,358 | 0.17 | 2019 | 9 |
| Antisocial behaviour^1^ | 16,400 | - | - | 0.052 | 2017 | 10 |
| **Negative controls** |  |  |  |  |  |  |
| Handedness (left vs. right) | 331,037 | 31,856 | 299,181 | 0.025 | 2019 | 11 |
| Cataracts | 127,603 | 11,986 | 115,617 | 0.019 | 2019 | 12 |

^1^These GWASs were based on quantitative traits in population-based cohorts.

^2^ALSPAC was removed from these GWAS summary statistics to avoid sample overlap

^3^We proposed to use these GWASs in our Stage 1 pre-registration; however, since then, new, larger GWASs were published for bipolar disorder and antisocial behaviour, allowing us to benefit from greater power. In addition, the original antisocial behaviour GWAS^10^ included ALSPAC, so could have introduced bias.

The Total N does not refer to the effective N.

# Supplementary Table 6. ACE definitions and measures in the ALSPAC cohort.

| ACE | Definition | Informant | Assessment type | Exposure period | Assessment  phase(s) | Criteria for presence of the ACE |
| --- | --- | --- | --- | --- | --- | --- |
| Maltreatment | Any of the following: sexual abuse, physical abuse (parent/partner was physically cruel to child), emotional abuse (parent/partner was emotionally cruel to child), or neglect (child always feels left out of things; never understood by parents) | Parent, child | Questionnaires | 0-9y | 8wk, 8m, 1y 6m, 1y 9m, 2y 6m, 2y 9m, 3y 6m, 3y 11m, 4y 9m, 5y 1m, 5y 9m, 6y 1m, 6y 9m, 8y 1m, 8y 9m, 9y 2m, 9y 7m | Reported at least once by age 9 |
| Domestic violence | Parents kicked, bitten or hit each other; physically twisted arm; throw(n) bodily; beaten each other up; choked or strangled each other; threatened each other with knife; used knife or other weapon on each other | Parent | Questionnaires | 0-9y | 8m, 1y 9m, 2y 9m, 3y 11m, 5y 1m, 6y 1m, 8y 1m, 9y 2m | Reported at least once by age 9 |
| Parental mental illness | Parent has hurt themselves on purpose, attempted suicide; taken medication for anxiety or depression; Edinburgh Postnatal Depression Scale (EPDS) score >12; diagnosed with schizophrenia, bulimia, or anorexia nervosa | Parent | Questionnaires | 0-9y | 8w, 8m, 1y 9m, 2y 9m, 3y 11m, 5y 1m, 6y 1m, 8y 1m, 9y 2m | Reported at least once by age 9 |
| Parent substance abuse | Parent smoked cannabis daily; used hard drugs (including crack, heroin, amphetamine, opiate, cocaine, methadone, meth), had a recent hard drug addiction; had Alcoholism/ drink problem; Alcohol Use Disorders Identification Test (AUDIT) score>8 | Parent | Questionnaires | 0-9y | 8wk, 8m, 1y 9m, 2y 9m, 3y 11m, 5y 1m, 6y 1m, 8y 1m, 9y 2m | Reported at least once by age 9 |
| Parental criminality | Parent had a court conviction; convicted of an offence | Parent | Questionnaires | 0-9y | 8w, 8m, 1y 9m, 2y 9m, 3y 11m, 5y 1m, 6y 1m, 9y 2m | Reported at least once by age 9 |
| Parental separation | Parents separated or divorced | Parent | Questionnaires | 0-9y | 8w, 8m, 1y 9m, 2y 9m, 3y 11m, 5y 1m, 6y 1m, 8y 1m, 9y 2m | Reported at least once by age 9 |

Acronyms: ACE = adverse childhood experience; wk = weeks; m = months; y = years

# Supplementary Table 7. Items from the Development and Wellbeing Assessment (DAWBA) used to derive mental health measures in ALSPAC.

| **Module** | **Variable** | **Label** |
| --- | --- | --- |
| Separation anxiety | kv4000 | D1a: Child is particularly attached to Mother or Mother figure |
|  | kv4020 | D3a: In past month child has often worried about something unpleasant happening to or losing special person |
|  | kv4021 | D3b: In past month child has often worried about being taken away from special person |
|  | kv4022 | D3c: In past month child has not wanted to go to school in case something bad happened to special person |
|  | kv4023 | D3d: In past month child has worried about sleeping alone |
|  | kv4024 | D3e: In past month child has left bed at night to check on or to sleep near special person |
|  | kv4025 | D3f: In past month child has worried about sleeping in a strange place |
|  | kv4026 | D3g: In past month child has been afraid to be alone in room without special person |
|  | kv4027 | D3h: In past month child has had bad dreams about separation from special person |
|  | kv4028 | D3i: In past month child has felt ill when had to leave special person |
|  | kv4029 | D3j: In past month child has become upset at being apart from special person |
| Social anxiety | kv5010 | F2a: In past month child has been afraid of meeting new people |
|  | kv5011 | F2b: In past month child has been afraid of meeting a lot of people |
|  | kv5012 | F2c: In past month child has been afraid of speaking in class |
|  | kv5013 | F2d: In past month child has been afraid of reading out loud in front of others |
|  | kv5014 | F2e: In past month child has been afraid of writing in front of others |
|  | kv5015 | F2f: In past month child has been afraid of eating in front of others |
| Generalised anxiety | kv6500 | J1: Child worries |
|  | kv6510 | J2: Child has had other worries in past 6 months that have interfered with life |
|  | kv6520 | J3a: Child worries about past behaviour |
|  | kv6521 | J3b: Child worries about school work |
|  | kv6522 | J3c: Child worries about disasters |
|  | kv6523 | J3d: Child worries about own health |
|  | kv6524 | J3e: Child worries about bad things happening to others |
|  | kv6525 | J3f: Child worries about the future |
|  | kv6526 | J3g: Child worries about other things |
|  | kv6550 | J6a: Worries lead to child being restless, tense or on edge |
|  | kv6551 | J6b: Worries lead to child being easily tired |
|  | kv6552 | J6c: Worries lead to difficulties in concentrating |
|  | kv6553 | J6d: Worries lead to irritability |
|  | kv6554 | J6e: Worries lead to child looking tense |
|  | kv6555 | J6f: Worries interfere with sleep |
| Major depression | kv7000 | K1: In past month child has been very sad |
|  | kv7010 | K2: In past month child has been grumpy or irritable in a way that was out of character |
|  | kv7020 | K4: In past month there have been times when child lost interest in everything |
|  | kv7031 | K5a: In past month child seemed tired all the time |
|  | kv7032 | K5b: In past month child was eating much more or less |
|  | kv7033 | K5c: In past month child either lost or gained a lot of weight |
|  | kv7034 | K5d: In past month child found sleeping hard |
|  | kv7035 | K5e: In past month child slept too much |
|  | kv7036 | K5f: In past month there was a period when child was agitated or restless |
|  | kv7037 | K5g: In past month there was a period when child felt worthless or unnecessarily guilty |
|  | kv7038 | K5h: In past month there was a period when child found it hard to concentrate |
|  | kv7039 | K5i: In past month child thought about death a lot |
|  | kv7040 | K5j: In past month child talked about harming or killing themselves |
|  | kv7041 | K5k: In past month child tried to harm or kill themselves |
|  | kv7042 | K5l: Child has ever tried to harm or kill self |
| Hyperkinesis/ ADHD | kv7510 | L2a: In last 6 months child often fidgets |
|  | kv7511 | L2b: In last 6 months child found it hard to sit still for long |
|  | kv7512 | L2c: In last 6 months child ran or climbed around when should not |
|  | kv7513 | L2d: In last 6 months child found it hard to take part in activities without making noise |
|  | kv7514 | L2e: In last 6 months child found it hard to calm down when asked to |
|  | kv7520 | L3a: In last 6 months child blurted out answers before hearing questions properly |
|  | kv7521 | L3b: In last 6 months child found it hard to wait for turn |
|  | kv7522 | L3c: In last 6 months child often butted in on others conversations or games |
|  | kv7523 | L3d: In last 6 months child often went on talking even if told to stop or no one is listening |
|  | kv7530 | L4a: In last 6 months child makes careless mistakes o’r doesn't pay attention |
|  | kv7531 | L4b: In last 6 months child seems to lose interest in what child is doing |
|  | kv7532 | L4c: In last 6 months child does not listen to people |
|  | kv7533 | L4d: In last 6 months child often does not complete jobs |
|  | kv7534 | L4e: In last 6 months child has had difficulty organising themselves |
|  | kv7535 | L4f: In last 6 months child often tried to avoid things involving thought |
|  | kv7536 | L4g: In last 6 months child often lost things needed for school |
|  | kv7537 | L4h: In last 6 months child has been easily distracted |
|  | kv7538 | L4i: In last 6 months child was often forgetful |
| Conduct/ oppositional disorders | kv8000 | M1: Mothers assessment of how child's awkward behaviour compares with other children |
|  | kv8010 | M2a: In past 6 months child has had severe tantrums |
|  | kv8011 | M2b: In past 6 months child has argued with grown-ups |
|  | kv8012 | M2c: In past 6 months child has taken no notice of rules or refused to do as told |
|  | kv8013 | M2d: In past 6 months child has done things to annoy others |
|  | kv8014 | M2e: In past 6 months child has blamed others for own mistakes |
|  | kv8015 | M2f: In past 6 months child has been touchy or easily annoyed |
|  | kv8016 | M2g: In past 6 months child has been angry or resentful |
|  | kv8017 | M2h: In past 6 months child has been spiteful |
|  | kv8018 | M2i: In past 6 months child has tried to get own back on others |
|  | kv8080 | M9a: In past year child has told lies to get favours or to get out of things |
|  | kv8082 | m9b: in past year child has often started fights |
|  | kv8084 | M9c: In past year child has bullied or threatened people |
|  | kv8086 | m9d: in past year child has stayed out much later than was supposed to |
|  | kv8088 | M9e: In past year child has stolen things |
|  | kv8090 | m9f: in past year child has run away from home or stayed out all night without p |
|  | kv8092 | M9g: In past year child has often played truant |

# Supplementary Table 8. Genotyping and quality control procedures.

|  | **ALSPAC** | **ABCD** | **CATSS^2^** |
| --- | --- | --- | --- |
| **Genotyping platform** | Illumina HumanHap550 | Affymetrix NIDA Smokescreen Array | Illumina Infinium PsychArray-24 Beadchip and the Illumina Infinium Global Screening Array-24 BeadChip |
| **QC: Sample exclusion** |  |  |  |
| Low call rate | >3% | >10% (saliva); >20% (blood)*^1^ | >2% |
| Outlying heterozygosity | > 3 standard deviations from the mean | Autosomal inbreeding coefficient F > 0.2* | Autosomal inbreeding coefficient F outside +/- 0.2 |
| Relatedness | IBD > 0.1875 | IBD ≥ 0.20 | Twin-based sample; analysis will take twin relatedness into account |
| Gender mismatches | X-chromosome F <= 0.8 for male or >= 0.2 for female | X-chromosome F < 0.5 for male or > 0.5 for female* | X-chromosome F <= 0.9 for male or >= 0.5 for female |
| Ancestral outliers | Non-European ancestry (self-report); samples that clustered outside the CEU HapMap population using multidimensional scaling of genome-wide IBS [identity by state] pairwise distances. | Non-European ancestry | Non-European ancestral outliers > 6 standard deviations from the mean values of the first two principal components in 1000-Genomes European populations. |
| **QC: SNP exclusion** |  |  |  |
| Low call rate | > 5% | > 10%* | > 2% |
| Deviation from Hardy-Weinberg equilibrium | HWE P < 5E-7 | HWE P < 1e-6 | HWE P < 1e-6 (MZ twins) and P < 1e-10 (DZ twins) |
| Non-autosomal SNPs | Excluded non-autosomal SNPs | Excluded non-autosomal SNPs | Excluded non-autosomal SNPs |
| Minor allele frequency | < 1% | < 1% | < 1% |
| **Imputation panel** | HRC panel | TOPMed panel | HRC panel |
| **Principal components** | 10 PCs, based on a subset of LD independent SNPs | 10 PCs, based on a subset of LD independent SNPs | 10 PCs, based on a subset of LD independent SNPs |

Note. QC = quality control; HRC = Haplotype Reference Consortium. * = Performed already as part of quality control by the ABCD Data Analysis, Informatics & Resource Center. ^1^ The ABCD Study team used different criteria to filter SNPs for low call rate depending on whether genotype was measured in blood or saliva, because more stringent call rate filtering resulted in a different number of SNPs between saliva and blood samples. ^2^We did not use the CATSS dataset due to access issues but show the planned quality control procedures for CATSS for transparency.

# Supplementary Table 9. ACE definitions and measures in the ABCD cohort.

| ACE | Definition | Informant | Assessment type | Exposure period | Assessment  phase | Criteria for presence of the ACE |
| --- | --- | --- | --- | --- | --- | --- |
| Maltreatment | Sexual abuse (was touched by/touched an adult, did sexual things with an adult, or was forced by a peer into sexual activities), physical abuse (shot, stabbed, or beaten brutally by an adult in the home, beaten to the point of having bruises by an adult in the home), emotional abuse (threatened to be killed by a family member or non-family member), or emotional neglect (≥ 2 of the following: caregiver does not believe in showing love for the child; does not make child feel better when upset; or when talking over worries; is not easy to talk to; does not smile at child very often) | Parent, child | Questionnaires (KSADS-PTSD; CRPBI) | 0-9/10y | Baseline | Any form of maltreatment reported by parent or child |
| Domestic violence | Child witnessed adults in the home push, shove, or hit each other; family members hit each other | Parent, child | Questionnaires (KSADS-PTSD; FES) | 0-9/10y | Baseline | Any instance of domestic violence reported by parent or child |
| Parental mental illness | Parent attempted or died by suicide; experienced a depressive episode; ASR score > 63 for depression, anxiety, or ADHD; had psychotic experiences (visions, hallucinations, or paranoia) for ≥6 months; had manic episode | Parent | Questionnaires (FAS; ASR) | 0-9/10y | Baseline | Any instance of parental mental illness reported |
| Parent substance abuse | Parent used drugs for non-medical purposes multiple times weekly in the past 6 months; parent had problems due to alcohol or drug use, including: arrests or DUIs; harm to health; in an alcohol/drugs treatment program; marital separation or divorce; laid off or fired from work; isolated self from family, caused arguments or were drunk/high a lot | Parent | Questionnaires (FAS; ASR) | 0-9/10y | Baseline | Parent used drugs multiple times weekly or had any aforementioned problem due to alcohol or drug use |
| Parental criminality | Parent got in trouble with the law, or went to jail, family member arrested | Parent | Questionnaire (ALES) | 0-9/10y | 1y follow-up | Any instance of parental criminality that was reported to occur by age 9/10y (i.e., not in the year between baseline and 1-year follow-up) |
| Parental separation | Parents separated or divorced; parent’s current partner is not the child’s biological or adoptive parent | Parent | Questionnaire (Demographic Survey) [baseline]; Parent Life Survey [1-year follow-up]) | 0-9/10y | Baseline; 1y follow-up | Parental separation that was reported to occur by age 9/10y (i.e., not between baseline and 1-year follow-up) |

Acronyms: ACE = adverse childhood experience; y = years; KSADS-PTSD = Kiddie Schedule for Affective Disorders and Schizophrenia – PTSD module; CRPBI = Children’s Report of Parent Behavior Inventory; FES = Family Environment Scale (Family Conflict subscale); ASR = Adult Self Report; FAS = Family History Assessment Module; ALES = Adverse Life Events Scale

# Supplementary Table 10. Items from the CBCL used to derive mental health measures in the ABCD Study.

| **Subscale** | **Variable** | **Label** |
| --- | --- | --- |
| Anxious/depressed | cbcl_q14_p | Cries a lot |
|  | cbcl_q29_p | Fears certain animals, situations, or places, other than school |
|  | cbcl_q30_p | Fears going to school |
|  | cbcl_q31_p | Fears he/she might think or do something bad |
|  | cbcl_q32_p | Feels he/she has to be perfect |
|  | cbcl_q33_p | Feels or complains that no one loves him/her |
|  | cbcl_q35_p | Feels worthless or inferior |
|  | cbcl_q45_p | Nervous, highstrung, or tense |
|  | cbcl_q50_p | Too fearful or anxious |
|  | cbcl_q52_p | Feels too guilty |
|  | cbcl_q71_p | Self-conscious or easily embarrassed |
|  | cbcl_q91_p | Talks about killing self |
|  | cbcl_q112_p | Worries |
| Withdrawn/depressed | cbcl_q05_p | There is very little he/she enjoys |
|  | cbcl_q42_p | Would rather be alone than with others |
|  | cbcl_q65_p | Refuses to talk |
|  | cbcl_q69_p | Secretive, keeps things to self |
|  | cbcl_q75_p | Too shy or timid |
|  | cbcl_q102_p | Underactive, slow moving, or lacks energy |
|  | cbcl_q103_p | Unhappy, sad, or depressed |
|  | cbcl_q111_p | Withdrawn’, doesn't get involved with others |
| Somatic complaints | cbcl_q47_p | Nightmares |
|  | cbcl_q49_p | Constipated, doesn't move bowels |
|  | cbcl_q51_p | Feels dizzy or lightheaded |
|  | cbcl_q54_p | Overtired without good reason |
|  | cbcl_q56a_p | Aches or pains (not stomach or headaches) |
|  | cbcl_q56b_p | Headaches |
|  | cbcl_q56c_p | Nausea, feels sick |
|  | cbcl_q56d_p | Problems with eyes (not if corrected by glasses) |
|  | cbcl_q56e_p | Rashes or other skin problems |
|  | cbcl_q56f_p | Stomach aches |
|  | cbcl_q56g_p | Vomiting, throwing up |
| Rule-breaking behaviour  Rule-breaking behaviour (continued) | cbcl_q02_p | Drinks alcohol without parents' approval |
|  | cbcl_q26_p | Doesn't seem to feel guilty after misbehaving |
|  | cbcl_q28_p | Breaks rules at home, school or elsewhere |
|  | cbcl_q39_p | Hangs around with others who get in trouble |
|  | cbcl_q43_p | Lying or cheating |
|  | cbcl_q63_p | Prefers being with older kids |
|  | cbcl_q67_p | Runs away from home |
|  | cbcl_q72_p | Sets fires |
|  | cbcl_q73_p | Sexual problems |
|  | cbcl_q81_p | Steals at home |
|  | cbcl_q82_p | Steals outside the home |
|  | cbcl_q90_p | Swearing or obscene language |
|  | cbcl_q96_p | Thinks about sex too much |
|  | cbcl_q99_p | Smokes, chews, or sniffs tobacco |
|  | cbcl_q101_p | Truancy, skips school |
|  | cbcl_q105_p | Uses drugs for non medical purposes (don't include alcohol or tobacco) |
|  | cbcl_q106_p | Vandalism |
| Aggressive behaviour | cbcl_q03_p | Argues a lot |
|  | cbcl_q16_p | Cruelty, bullying, or meanness to others |
|  | cbcl_q19_p | Demands a lot of attention |
|  | cbcl_q20_p | Destroys his/her own things |
|  | cbcl_q21_p | Destroys things belonging to his/her family or others |
|  | cbcl_q22_p | Disobedient at home |
|  | cbcl_q23_p | Disobedient at school |
|  | cbcl_q37_p | Gets in many fights |
|  | cbcl_q57_p | Physically attacks people |
|  | cbcl_q68_p | Screams a lot |
|  | cbcl_q86_p | Stubborn, sullen, or irritable |
|  | cbcl_q87_p | Sudden changes in mood or feelings |
|  | cbcl_q88_p | Sulks a lot |
|  | cbcl_q89_p | Suspicious |
|  | cbcl_q94_p | Teases a lot |
|  | cbcl_q95_p | Temper tantrums or hot temper |
|  | cbcl_q97_p | Threatens people |
|  | cbcl_q104_p | Unusually loud |
| Attention problems | cbcl_q01_p | Acts too young for his/her age |
|  | cbcl_q04_p | Fails to finish things he/she starts |
|  | cbcl_q08_p | Can't concentrate, can't pay attention for long |
|  | cbcl_q10_p | Can't sit still, restless, or hyperactive |
|  | cbcl_q13_p | Confused or seems to be in a fog |
|  | cbcl_q17_p | Daydreams or gets lost in his/her thoughts |
|  | cbcl_q41_p | Impulsive or acts without thinking |
|  | cbcl_q61_p | Poor school work |
|  | cbcl_q78_p | Inattentive or easily distracted |
|  | cbcl_q80_p | Stares blankly |

# Supplementary Table 11. Method for estimating each original parameter included in the genetic sensitivity correlation matrix.

| Parameter | Method of estimating parameter |
| --- | --- |
| Correlation between the ACE and the mental health outcome | 1. Run a linear regression model predicting the mental health outcome from the ACE |
|  | 2. Take the square root of the R^2^ value reflecting the variance in the mental health outcome explained by the ACE |
| Correlation between observed polygenic scores for mental health problems and the ACE (*a* path) | 1. Run a probit regression model predicting the ACE from the polygenic scores |
|  | 2. Take the square root of the R^2^ value reflecting the variance in the (latent-response) ACE variable explained by the polygenic scores |
| Correlation between observed polygenic scores and the mental health outcome (*b* path) | 1. Run a linear regression model predicting the mental health outcome from the polygenic scores for mental health problems |
|  | 2. Take the square root of the R^2^ value reflecting the variance in the mental health outcome explained by the polygenic scores |

Note. All models were estimated using the *lavaan* package^36^, accounting for sex and principal components.

# Supplementary Table 12. Auxiliary variables included in the ALSPAC imputation model.

| Auxiliary variable | Age assessed | Informant |
| --- | --- | --- |
| *Early sociodemographic indicators* |  |  |
| Child sex | Birth | Mother |
| Child ethnicity | 32 weeks | Mother |
| Household social class | 18 weeks, 32 weeks | Mother |
| Maternal age at birth | 8 weeks | Mother |
| Mother’s home ownership status | 8 weeks | Mother |
| Parity | 18 weeks | Mother |
| Maternal marital status at birth | 8 weeks | Mother |
| Mother and partner’s highest educational qualification | 18 and 32 weeks | Mother, partner |
| Birthweight | Birth | Records |
| Gestational age | Birth | Records |
| Maternal weight | 8 weeks | Mother |
| Maternal BMI | 8 weeks | Mother |
| Maternal smoking during pregnancy | 18 weeks, 32 weeks | Mother |
| *Adversity exposure before birth* |  |  |
| Mother became homeless | 8 weeks | Mother |
| Maternal Edinburgh Postnatal Depression Scale (EPDS) score | 18 and 32 weeks | Mother |
| Mother’s partner’s EPDS score | 18 weeks | Partner |
| Mother taking medication for depression | 8 months | Mother |
| Mother’s opinion of neighbourhood | 8 weeks | Mother |
| Partner of mother was convicted of offence | 8 weeks | Mother |
| Mother separated from partner | 8 weeks | Mother |
| Partner’s hard drug use | 18 weeks | Mother |
| Mother had difficulty affording heat or food | 32 weeks | Mother |
| *Adversity exposure in adolescence* |  |  |
| Physical abuse | 10-18 years | Parent, self |
| Sexual abuse | 10-18 years | Parent, self |
| Emotional abuse | 10-18 years | Parent, self |
| Emotional neglect | 10-18 years | Parent, self |
| Domestic violence | 10-18 years | Parent, self |
| Parental substance abuse | 10-18 years | Parent |
| Parental mental illness | 10-18 years | Parent, self |
| Parental criminality | 10-18 years | Parent, self |
| Parental separation | 10-18 years | Parent, self |
| *Alternative forms of adversity* |  |  |
| Bullying victimisation | 8-18 years | Self |
| Parent-child bond | 0-18 years | Parent, self |
| Financial difficulties | 0-18 years | Parent |
| Low social class | 0-8 years | Parent |
| *Adversity exposure in young adulthood* |  |  |
| Mother’s partner was emotionally cruel to child | 22 years | Self |
| Maternal antidepressant use | 18.5 years | Mother |
| Maternal EPDS score | 18.5 years | Mother |
| Mother separated from partner | 18.5 years | Mother |
| Partner of child was violent towards child | 22 years | Self |
| Partner of child sexually abused child | 22 years | Self |
| Partner of child emotionally abused child | 22 years | Self |
| Parent’s alcohol use disorders identification score (AUDIT) | 18.5 years | Mother |
| *DAWBA measures* |  |  |
| Internalising problems subscales | 7, 10, and 12 years | Mother |
| Externalising problems subscales | 7, 10, and 12 years | Mother |

# Supplementary Table 13. Details of how variables were included in the ALSPAC imputation model.

| Variable to be included in the imputation model | Type of variable | How variable will be transformed in the imputation model* |
| --- | --- | --- |
| *Exposure: ACEs (0-9 years)* |  |  |
| Maltreatment variables: |  |  |
| Physical abuse | Dichotomous | Noms |
| Sexual abuse | Dichotomous | Noms |
| Emotional abuse | Dichotomous | Noms |
| Emotional neglect | Dichotomous | Noms |
| Domestic violence | Dichotomous | Noms |
| Parental substance abuse | Dichotomous | Noms |
| Parental mental illness | Dichotomous | Noms |
| Parental criminality | Dichotomous | Noms |
| Parental separation | Dichotomous | Noms |
| *Outcome: DAWBA (10 years)* |  |  |
| Internalising problems | Continuous | Continuous |
| Externalising problems | Continuous | Continuous |
| *Early sociodemographic indicators* |  |  |
| Sex | Dichotomous | Noms |
| Parental social class during pregnancy | Categorical (6) | Ords |
| Child ethnicity | Dichotomous | Noms |
| Maternal age at birth | Continuous | Continuous |
| Mother’s home ownership during pregnancy | Categorical (7) | Noms |
| Parity | Continuous | Continuous |
| Maternal marital status at birth | Categorical (6) | Noms |
| Mother’s highest educational level (mother-reported) | Categorical (5) | Ords |
| Partner’s highest educational level (mother-reported) | Categorical (5) | Ords |
| Mother’s highest educational level (partner-reported) | Categorical (5) | Ords |
| Partner’s highest educational level (partner-reported) | Categorical (5) | Ords |
| Birthweight | Continuous | Continuous |
| Gestational age | Continuous | Continuous |
| Maternal pre-pregnancy weight (kg) | Continuous | Continuous |
| Maternal pre-pregnancy BMI | Continuous | Continuous |
| Maternal smoking during the 1st trimester | Dichotomous | Noms |
| Maternal smoking during the 2nd trimester | Dichotomous | Noms |
| Maternal smoking during the 3rd trimester (prospectively reported) | Dichotomous | Noms |
| Maternal smoking during the 3rd trimester (retrospectively reported) | Dichotomous | Noms |
| *Adversity exposure before birth* |  |  |
| Mother became homeless | Categorical (2) | Noms |
| Maternal depression score (EPDS) at 18 wks gestation | Continuous | Continuous |
| Partner EPDS score at 18 wks gestation | Continuous | Continuous |
| Maternal EPDS score at 32 wks gestation | Continuous | Continuous |
| Antidepressant use by mother | Dichotomous | Noms |
| Difficulty affording food during pregnancy | Categorical (4) | Ords |
| Difficulty affording heating during pregnancy | Categorical (4) | Ords |
| Mother’s opinion of neighbourhood during pregnancy | Categorical (4) | Ords |
| Partner convicted of an offence during pregnancy | Categorical (2) | Noms |
| Partner separated since pregnancy | Dichotomous | Noms |
| Mother divorced since pregnancy | Dichotomous | Noms |
| Partner hard drug use during pregnancy | Categorical (2) | Noms |
| *Adversity exposure in adolescence (10-18 years)* | |  |
| Physical abuse | Dichotomous | Noms |
| Sexual abuse | Dichotomous | Noms |
| Emotional abuse | Dichotomous | Noms |
| Emotional neglect | Dichotomous | Noms |
| Domestic violence | Dichotomous | Noms |
| Parental substance abuse | Dichotomous | Noms |
| Parental mental illness | Dichotomous | Noms |
| Parental criminality | Dichotomous | Noms |
| Parental separation | Dichotomous | Noms |
| *Other forms of adversity (0-18 years)* | |  |
| Bullying victimisation | Dichotomous | Noms |
| Parent-child bond | Dichotomous | Noms |
| Financial difficulties | Dichotomous | Noms |
| Low social class | Dichotomous | Noms |
| *Adversity exposure in young adulthood (18-21 years)* | |  |
| Mother’s partner was emotionally cruel to child | Dichotomous | Noms |
| Antidepressant use by mother when child was 18yrs | Dichotomous | Noms |
| Maternal EPDS score when child was 18yrs | Continuous | Continuous |
| Mother separated from partner when child was 18yrs | Dichotomous | Noms |
| Maternal AUDIT score when child was 18yrs | Continuous | Continuous |
| Paternal AUDIT score when child was 18yrs | Continuous | Continuous |
| Participant’s partner used physical force | Dichotomous | Noms |
| Participant’s partner used more severe physical force | Dichotomous | Noms |
| Participant’s partner pressured them into kissing/touching | Dichotomous | Noms |
| Participant’s partner physically forced them into kissing/touching | Dichotomous | Noms |
| Participant’s partner pressured them into sexual intercourse | Dichotomous | Noms |
| Participant’s partner physically forced them into sexual intercourse | Dichotomous | Noms |
| Participant’s partner made them feel scared of frightened | Dichotomous | Noms |
| *DAWBA (7- 13 years)* |  |  |
| Internalising problems at age 7 | Continuous | Continuous |
| Externalising problems at age 7 | Continuous | Continuous |
| Internalising problems at age 13 | Continuous | Continuous |
| Externalising problems at age 13 | Continuous | Continuous |

DAWBA= Development and Well-being Assessment. ***** The *Amelia* package^3^ requires data to be distributed as multivariate normal; hence transformations are required for non-normally distributed variables. Continuous variables were assessed for skewness, and any skewed variables were transformed using a log-linear transformation (“logs”). This table was adapted from Houtepen and colleagues^1^ in their data note on dealing with missingness in ACE measures

# Supplementary Table 14. Details of variables included in the ABCD imputation model.

| **Variable to be included in the imputation model** | **Type of variable** | **How variable will be transformed in the imputation model*** |
| --- | --- | --- |
| ***Exposure: ACEs (0-9/10 years)*** |  |  |
| Maltreatment variables: |  |  |
| Physical abuse | Dichotomous | Noms |
| Sexual abuse | Dichotomous | Noms |
| Emotional abuse | Dichotomous | Noms |
| Emotional neglect | Dichotomous | Noms |
| Domestic violence | Dichotomous | Noms |
| Parental substance abuse | Dichotomous | Noms |
| Parental mental illness | Dichotomous | Noms |
| Parental criminality | Dichotomous | Noms |
| Parental separation | Dichotomous | Noms |
| ***Outcome: CBCL (9/10 years)*** |  |  |
| Internalising problems | Continuous | Continuous |
| Externalising problems | Continuous | Continuous |
| ***Sociodemographic indicators*** |  |  |
| Child sex | Dichotomous | Noms |
| Child race | Categorical | Noms |
| Parental employment | Categorical | Noms |
| Parental highest education qualification | Categorical | Noms |
| Family income | Categorical | Noms |
| Household size | Continuous | Noms |
| Maternal age at birth | Continuous | Noms |
| ***Adversity exposure before birth*** |  |  |
| Birthweight | Continuous | Continuous |
| Premature birth | Dichotomous | Noms |
| Maternal smoking during pregnancy | Dichotomous | Noms |
| Maternal alcohol consumption during pregnancy | Dichotomous | Noms |
| Pregnancy complications | Dichotomous | Noms |
| ***Other forms of adversity*** |  |  |
| Difficulty affording food | Dichotomous | Noms |
| Difficulty affording gas/electricity/oil | Dichotomous | Noms |
| Evicted from home because could not pay rent/mortgage | Dichotomous | Noms |
| Bullying victimisation | Dichotomous | Noms |
| Parent AESBA mental health scores: |  |  |
| Anxious/Depressed scale | Continuous | Continuous |
| Withdrawn scale | Continuous | Continuous |
| Somatic Complaints scale | Continuous | Continuous |
| Thought Problems scale | Continuous | Continuous |
| Attention Problems scale | Continuous | Continuous |
| Aggressive Behaviour scale | Continuous | Continuous |
| Rule-Breaking Behaviour scale | Continuous | Continuous |

***** The *Amelia* package^3^ requires data to be distributed as multivariate normal; hence transformations are required for non-normally distributed variables. Noms=nominal variables

# Supplementary Table 15. ACE definitions and measures in the CATSS cohort.

| ACE | Definition | Informant^1^ | Assessment type | Exp. period | Criteria for presence of the ACE |
| --- | --- | --- | --- | --- | --- |
| Maltreatment | Any of the following: sexual abuse (forced/threatened into being touched/touching someone, or having sex [oral, anal, genital] by someone else), physical abuse (hit, choked, burned, beaten or severely punished, or Patient Register record of injury caused by interpersonal violence), emotional abuse or neglect (frequently shamed, embarrassed, ignored, or repeatedly told that he/she were “no good”), or physical neglect (child is not fed, not properly clothed, or left to take care of him/herself) | Parent; National Patient Register (for physical abuse) | Interview (LSC-R); population-based register (for physical abuse) | 0-9y | Any of the following: reported by parent or ICD-10 code of X85-X99 or Y00-Y09 in the Patient Register (for physical abuse)^15^ |
| Domestic violence | Child witnessed physical violence (kicked, hit, punched or beaten up) between family members; child's parent or other adult living with the child was arrested for intimate partner violence or general domestic violence | Parent; National Crime Register | Interview (LSC-R); population-based registers | 0-9y | Any of the following: reported by parent, code of 0411, 0412, 0422, 0423, 0424, 0425, 0440, 0441, 0442, and 0443 in the National Crime Register^37^ |
| Parental mental illness | Parent received diagnosis of anorexia nervosa, anxiety disorder, bulimia, bipolar disorder, depression, schizophrenia, or suicide attempt/self-injury in inpatient or outpatient visit; received psychiatric medication (e.g., antidepressant, antipsychotic, benzodiazepine, mood stabiliser) | National Patient Register; Prescribed Drug Register | Population-based registers | 0-9y | Any of the following: Patient Register ICD-10 code of F50.0-F50.1^38^, F40-42, F44-45, F48, F50.2-F50.3^15^, F30-31, F32-F39 except F32.2, F20, X60-X84, Y10-Y34; Prescribed Drug Register ATC code of N06A, N05A, N05BA^39,40^, N03AG01, N03AX09, N03AF01, N03AF02, N05AN01^39^ |
| Parental substance abuse | Substance abuse in household; medical records of parent having mental and behavioural disorders due to alcohol and drugs or poisonings, alcohol/drug poisoning, somatic illnesses caused by alcohol and drugs, alcohol/drug involvement, and parent convictions (or suspicions after a completed investigation by police, customs authority, or the prosecution service) of alcohol or drug-related crimes, or driving under the influence of alcohol or illicit substances | Parent; National Patient Register; National Crime Register; Register of Persons Suspected of Offenses | Interview (LSC-R); population-based registers | 0-9y | Any of the following: reported by parent; Patient Register ICD-10 code of F10-F19 (except x.5) G32.2, G62.1, G72.1, I42.6, K29.2, K70, K85, X41-2, X45, X61-2, X65, Y11 [with T43.6], Y12 [with T40], and Y15 [with T51],^41,42^ Crime Register ref. to SFS laws 1968:64 and law 1951:649, Persons Suspected of Offenses codes 3070, 510-512 |
| Parental criminality | Parent convicted of, or suspected of a crime after a completed investigation by police, customs authority, or the prosecution service | National Crime Register, Register of Persons Suspected of Offenses | Population-based registers | 0-9y | Any registration in the National Crime Register or the Register of Persons Suspected of Offenses^43^ |
| Parental separation | Parental divorce/separation; child not living with a parent previously lived with | Parent, National Statistics Tables, Total Population Register | Interview (LSC-R); population-based registers | 0-9y | Any of the following: reported by parent; divorce coded in ‘marriage status’ variable of Total Population Register^44^ |

Acronyms: ACE = adverse childhood experience; exp = exposure; LSC-R = Life Stressor Checklist-Revised^23^. ^1^ ‘Parent’ refers to parent response via telephone interview when CATSS participants were aged 9 years; the National Patient Register records all nationwide inpatient hospitalisations since 1987 and outpatient specialist visits since 2001; the Prescribed Drug Register records all prescription medications dispensed outside of hospitals since July 2005; the National Crime Register records all convictions in lower court from 1973, and the Register of Persons Suspected of Offenses records all incidents of individuals strongly suspected of crime since 1998

# Supplementary Table 16. Associations between ACEs and mental health after controlling for observed polygenic scores for mental health problems (complete cases).

|  | 1. **ALSPAC** | | | | 1. **ABCD** | | | |
| --- | --- | --- | --- | --- | --- | --- | --- | --- |
|  | **Total association** | **Adjusted association** | **Genetic confounding** | **Prop. genetically confounded** | **Total association** | **Adjusted association** | **Genetic confounding** | **Prop. genetically confounded** |
| **Internalising problems** | | | | | | | | |
| Maltreatment | 0.109 (0.079-0.139) | 0.104 (0.074-0.134) | 0.005 (0.002-0.008) | 0.044 (0.012-0.077) | 0.124 (0.097-0.152) | 0.123 (0.095-0.151) | 0.002 (-0.001-0.005) | 0.014 (-0.009-0.036) |
| Domestic violence | 0.061 (0.031-0.091) | 0.058 (0.027-0.088) | 0.003 (0.000-0.006) | 0.052 (0.000-0.104) | 0.093 (0.065-0.121) | 0.090 (0.061-0.118) | 0.003 (0.000-0.006) | 0.035 (0.002-0.068) |
| Parental mental illness | 0.179 (0.150-0.207) | 0.173 (0.144-0.202) | 0.006 (0.002-0.010) | 0.033 (0.011-0.056) | 0.276 (0.252-0.301) | 0.272 (0.247-0.296) | 0.005 (0.001-0.009) | 0.017 (0.004-0.031) |
| Parental substance abuse | 0.043 (0.013-0.074) | 0.039 (0.009-0.070) | 0.004 (0.001-0.007) | 0.088 (-0.007-0.183) | 0.140 (0.112-0.168) | 0.135 (0.107-0.163) | 0.005 (0.001-0.010) | 0.037 (0.006-0.068) |
| Parental criminality | 0.054 (0.024-0.085) | 0.056 (0.026-0.086) | -0.002 (-0.004-0.001) | -0.030 (-0.086-0.025)^X^ | 0.080 (0.051-0.108) | 0.077 (0.048-0.105) | 0.003 (0.000-0.006) | 0.041 (0.000-0.083) |
| Parental separation | 0.035 (0.004-0.065) | 0.033 (0.002-0.063) | 0.002 (-0.001-0.005) | 0.056 (-0.041-0.153) | 0.100 (0.072-0.128) | 0.094 (0.066-0.123) | 0.006 (0.001-0.010) | 0.056 (0.008-0.104) |
| **Externalising problems** | | | | | | | | |
| Maltreatment | 0.135 (0.106-0.165) | 0.131 (0.101-0.160) | 0.005 (0.000-0.009) | 0.034 (0.002-0.066) | 0.160 (0.133-0.188) | 0.156 (0.129-0.183) | 0.005 (0.001-0.009) | 0.029 (0.005-0.053) |
| Domestic violence | 0.083 (0.053-0.113) | 0.079 (0.049-0.109) | 0.004 (0.000-0.008) | 0.050 (0.001-0.099) | 0.157 (0.130-0.185) | 0.151 (0.124-0.178) | 0.006 (0.003-0.010) | 0.040 (0.016-0.065) |
| Parental mental illness | 0.151 (0.122-0.180) | 0.145 (0.116-0.174) | 0.006 (0.001-0.011) | 0.040 (0.008-0.072) | 0.276 (0.252-0.300) | 0.267 (0.243-0.292) | 0.010 (0.005-0.014) | 0.034 (0.018-0.050) |
| Parental substance abuse | 0.044 (0.013-0.074) | 0.038 (0.008-0.069) | 0.005 (0.001-0.009) | 0.120 (-0.003-0.242) | 0.183 (0.156-0.210) | 0.172 (0.145-0.199) | 0.012 (0.007-0.016) | 0.062 (0.035-0.089) |
| Parental criminality | 0.050 (0.020-0.081) | 0.054 (0.024-0.084) | -0.004 (-0.008-0.000) | -0.076 (-0.172-0.019)^X^ | 0.116 (0.088-0.144) | 0.109 (0.081-0.137) | 0.008 (0.004-0.012) | 0.065 (0.028-0.102) |
| Parental separation | 0.060 (0.030-0.091) | 0.054 (0.024-0.084) | 0.006 (0.002-0.010) | 0.105 (0.024-0.186) | 0.138 (0.110-0.165) | 0.125 (0.097-0.153) | 0.013 (0.008-0.018) | 0.094 (0.053-0.134) |

Note. Prop=proportion. Results are presented as standardized coefficients. ^X^ In ALSPAC, the proportion of the associations between parental criminality with internalising and externalising problems explained by polygenic scores was less than 0. This was because the proportion genetically confounded was calculated from a mediation model (see Extended Data Fig. 2**)** and the direct and indirect effects were in opposite directions.

# Supplementary Table 17*.* Associations between ACEs and mental health after controlling for latent polygenic scores capturing additional heritability in mental health problems (complete cases).

|  | **ALSPAC** | | | | **ABCD** | | | |
| --- | --- | --- | --- | --- | --- | --- | --- | --- |
|  | **Total association** | **Adjusted association** | **Genetic confounding** | **Prop. genetically confounded** | **Total association** | **Adjusted association** | **Genetic confounding** | **Prop. genetically confounded** |
| **Internalising problems** | | | | | | | | |
| Maltreatment | 0.109 (0.079-0.140) | 0.008 (-0.061-0.077) | 0.102 (0.046-0.158) | 0.929 (0.311-1.000) | 0.125 (0.096-0.153) | 0.058 (0.016-0.100) | 0.067 (0.040-0.094) | 0.537 (0.272-0.801) |
| Domestic violence | 0.065 (0.043-0.088) | 0.000 (NA- NA) ^X^ | 0.065 (0.043-0.088) | 1.000 (1.000-1.000) ^X^ | 0.093 (0.064-0.121) | 0.041 (0.003-0.080) | 0.051 (0.027-0.076) | 0.556 (0.235-0.877) |
| Parental mental illness | 0.182 (0.152-0.212) | 0.079 (0.013-0.145) | 0.103 (0.054-0.153) | 0.567 (0.252-0.883) | 0.287 (0.259-0.314) | 0.222 (0.186-0.259) | 0.065 (0.053-0.076) | 0.225 (0.168-0.283) |
| Parental substance abuse | 0.075 (0.055-0.095) | 0.000 (0.000-0.000) | 0.075 (0.055-0.095) | 1.000 (1.000-1.000) ^X^ | 0.138 (0.109-0.167) | 0.012 (-0.064-0.087) | 0.126 (0.066-0.187) | 0.916 (0.381-1.000) |
| Parental criminality | 0.068 (0.046-0.090) | 0.000 (NA- NA) ^X^ | 0.068 (0.046-0.090) | 1.000 (1.000-1.000) ^X^ | 0.100 (0.081-0.120) | 0.000 (NA-NA)^X^ | 0.100 (0.081-0.120) | 1.000 (1.000-1.000)^X^ |
| Parental separation | 0.058 (0.038-0.079) | 0.000 (NA- NA) ^X^ | 0.058 (0.038-0.079) | 1.000 (1.000-1.000) ^X^ | 0.110 (0.090-0.131) | 0.000 (0.000-0.000)^X^ | 0.110 (0.090-0.131) | 1.000 (1.000-1.000)^X^ |
| **Externalising problems** | | | | | | | | |
| Maltreatment | 0.137 (0.106-0.167) | 0.044 (-0.004-0.091) | 0.093 (0.060-0.126) | 0.681 (0.380-0.981) | 0.162 (0.133-0.190) | 0.094 (0.058-0.131) | 0.067 (0.046-0.089) | 0.417 (0.263-0.571) |
| Domestic violence | 0.083 (0.052-0.113) | 0.017 (-0.026-0.059) | 0.066 (0.038-0.095) | 0.800 (0.341-1.260) | 0.158 (0.130-0.186) | 0.107 (0.073-0.141) | 0.051 (0.031-0.070) | 0.321 (0.187-0.456) |
| Parental mental illness | 0.153 (0.123-0.184) | 0.043 (-0.011-0.096) | 0.110 (0.073-0.148) | 0.721 (0.410-1.032) | 0.286 (0.258-0.314) | 0.204 (0.168-0.241) | 0.082 (0.065-0.098) | 0.286 (0.214-0.357) |
| Parental substance abuse | 0.079 (0.059-0.098) | 0.000 (0.000-0.000) ^X^ | 0.079 (0.059-0.098) | 1.000 (1.000-1.000) ^X^ | 0.183 (0.155-0.212) | 0.064 (0.013-0.114) | 0.120 (0.086-0.153) | 0.652 (0.416-0.889) |
| Parental criminality | 0.067 (0.047-0.087) | 0.000 (NA- NA) ^X^ | 0.067 (0.047-0.087) | 1.000 (1.000-1.000) ^X^ | 0.116 (0.087-0.145) | -0.018 (-0.077-0.040) | 0.135 (0.090-0.179) | 1.000 (0.628-1.000) |
| Parental separation | 0.070 (0.049-0.091) | 0.000 (0.000-0.000) ^X^ | 0.070 (0.049-0.091) | 1.000 (1.000-1.000) ^X^ | 0.135 (0.106-0.164) | 0.001 (-0.057-0.059) | 0.135 (0.091-0.178) | 0.996 (0.567-1.000) |

Note. Prop=proportion. Results are presented as standardized coefficients. ^X^Confidence intervals could not be reliably computed and therefore these estimates should be interpreted with caution

# Supplementary References

1 Howard, D. M. *et al.* Genome-wide meta-analysis of depression identifies 102 independent variants and highlights the importance of the prefrontal brain regions. *Nat. Neurosci.* **22**, 343 (2019).

2 Purves, K. L. *et al.* A major role for common genetic variation in anxiety disorders. *Mol. Psychiatry*, doi:10.1038/s41380-019-0559-1 (2019).

3 Mullins, N. *et al.* Genome-wide association study of more than 40,000 bipolar disorder cases provides new insights into the underlying biology. *Nat. Genet.* **53**, 817-829 (2021).

4 Grove, J. *et al.* Identification of common genetic risk variants for autism spectrum disorder. *Nat. Genet.* **51**, 431-444 (2019).

5 Demontis, D. *et al.* Discovery of the first genome-wide significant risk loci for attention deficit/hyperactivity disorder. *Nat. Genet.* **51**, 63 (2019).

6 Tielbeek, J. J. *et al.* Uncovering the Genetic Architecture of Broad Antisocial Behavior through a Genome-Wide Association Study Meta-analysis. *bioRxiv*, 2021.2010.2019.462578, doi:10.1101/2021.10.19.462578 (2021).

7 Sanchez-Roige, S. *et al.* Genome-wide association study meta-analysis of the Alcohol Use Disorders Identification Test (AUDIT) in two population-based cohorts. *Am. J. Psychiatry* **176**, 107-118 (2019).

8 Pardiñas, A. F. *et al.* Common schizophrenia alleles are enriched in mutation-intolerant genes and in regions under strong background selection. *Nat. Genet.* **50**, 381 (2018).

9 Stahl, E. A. *et al.* Genome-wide association study identifies 30 loci associated with bipolar disorder. *Nat. Genet.* **51**, 793-803 (2019).

10 Tielbeek, J. J. *et al.* Genome-wide association studies of a broad spectrum of antisocial behavior. *JAMA psychiatry* **74**, 1242-1250 (2017).

11 De Kovel, C. G. & Francks, C. The molecular genetics of hand preference revisited. *Sci. Rep.* **9**, 1-9 (2019).

12 Watanabe, K. *et al.* A global overview of pleiotropy and genetic architecture in complex traits. *Nat. Genet.* **51**, 1339-1348 (2019).

13 Cheesman, R. *et al.* Childhood behaviour problems show the greatest gap between DNA-based and twin heritability. *Translational Psychiatry* **7**, 1284 (2017).

14 Anckarsäter, H. *et al.* The Child and Adolescent Twin Study in Sweden (CATSS). *Twin. Res. Hum. Genet.* **14**, 495-508 (2011).

15 Sariaslan, A., Arseneault, L., Larsson, H., Lichtenstein, P. & Fazel, S. Risk of Subjection to Violence and Perpetration of Violence in Persons With Psychiatric Disorders in Sweden. *JAMA Psychiatry*, doi:10.1001/jamapsychiatry.2019.4275 (2020).

16 Wolfe, J. & Kimerling, R. Gender issues in the assessment of posttraumatic stress disorder. in *Assessing psychological trauma and PTSD* (eds J. Wilson & T.M. Keane) 192-238 (Guilford, 1997).

17 Birmaher, B. *et al.* The screen for child anxiety related emotional disorders (SCARED): Scale construction and psychometric characteristics. *J. Am. Acad. Child Adolesc. Psychiatry* **36**, 545-553 (1997).

18 Angold, A. *et al.* Development of a short questionnaire for use in epidemiological studies of depression in children and adolescents. *Int. J. Methods Psychiatr. Res.* **6**, 237-249 (1995).

19 Birmaher, B. *et al.* Psychometric properties of the Screen for Child Anxiety Related Emotional Disorders (SCARED): a replication study. *J. Am. Acad. Child Adolesc. Psychiatry* **38**, 1230-1236 (1999).

20 Essau, C. A., Muris, P. & Ederer, E. M. Reliability and validity of the Spence Children's Anxiety Scale and the Screen for Child Anxiety Related emotional disorders in German children. *J. Behav. Ther. Exp. Psychiatry* **33**, 1-18 (2002).

21 Muris, P. *et al.* The screen for child anxiety related emotional disorders (SCARED) and traditional childhood anxiety measures. *J. Behav. Ther. Exp. Psychiatry* **29**, 327-339 (1998).

22 Rhew, I. C. *et al.* Criterion validity of the Short Mood and Feelings Questionnaire and one-and two-item depression screens in young adolescents. *Child and Adolescent Psychiatry and Mental Health* **4**, 8 (2010).

23 Turner, N., Joinson, C., Peters, T. J., Wiles, N. & Lewis, G. Validity of the Short Mood and Feelings Questionnaire in late adolescence. *Psychol. Assess.* **26**, 752 (2014).

24 Sharp, C., Goodyer, I. M. & Croudace, T. J. The Short Mood and Feelings Questionnaire (SMFQ): a unidimensional item response theory and categorical data factor analysis of self-report ratings from a community sample of 7-through 11-year-old children. *J. Abnorm. Child Psychol.* **34**, 365-377 (2006).

25 Taylor, M. J. *et al.* Association of genetic risk factors for psychiatric disorders and traits of these disorders in a Swedish population twin sample. *JAMA Psychiatry* **76**, 280-289 (2019).

26 Mårland, C. *et al.* The Autism–Tics, ADHD and other Comorbidities inventory (A-TAC): previous and predictive validity. *BMC Psychiatry* **17**, 403 (2017).

27 Larson, T. *et al.* Predictive properties of the A-TAC inventory when screening for childhood-onset neurodevelopmental problems in a population-based sample. *BMC Psychiatry* **13**, 233 (2013).

28 Larson, T. *et al.* The autism-tics, AD/HD and other comorbidities inventory (A-TAC): further validation of a telephone interview for epidemiological research. *BMC Psychiatry* **10**, 1 (2010).

29 Hansson, S. L. *et al.* Psychiatric telephone interview with parents for screening of childhood autism–tics, attention-deficit hyperactivity disorder and other comorbidities (A-TAC): preliminary reliability and validity. *The British Journal of Psychiatry* **187**, 262-267 (2005).

30 Purcell, S. *et al.* PLINK: a tool set for whole-genome association and population-based linkage analyses. *The American Journal of Human Genetics* **81**, 559-575 (2007).

31 Brikell, I. *et al.* The contribution of common genetic risk variants for ADHD to a general factor of childhood psychopathology. *Mol. Psychiatry*, 1, doi:10.1038/s41380-018-0109-2 (2018).

32 Lam, M. *et al.* RICOPILI: Rapid Imputation for COnsortias PIpeLIne. *Bioinformatics* **36**, 930-933 (2020).

33 Houtepen, L., Heron, J, Suderman, M, Tilling, K, Howe, L. Adverse childhood experiences in the children of the Avon Longitudinal Study of Parents and Children (ALSPAC). *Wellcome Open Research* **3** (2018).

34 Houtepen, L. C. *et al.* Associations of adverse childhood experiences with educational attainment and adolescent health and the role of family and socioeconomic factors: A prospective cohort study in the UK. *PLoS Med.* **17**, e1003031 (2020).

35 Honaker, J., King, G. & Blackwell, M. Amelia II: A Program for Missing Data. *Journal of Statistical Software* **45**, 1-47 (2010).

36 Rosseel, Y. Lavaan: An R package for structural equation modeling and more. Version 0.5–12 (BETA). *Journal of Statistical Software* **48**, 1-36 (2012).

37 Yu, R. *et al.* Mental disorders and intimate partner violence perpetrated by men towards women: A Swedish population-based longitudinal study. *PLoS Med.* **16** (2019).

38 Yao, S. *et al.* Familial Liability for Eating Disorders and Suicide Attempts: Evidence From a Population Registry in Sweden. *JAMA Psychiatry* **73**, 284-291, doi:10.1001/jamapsychiatry.2015.2737 (2016).

39 Fazel, S., Zetterqvist, J., Larsson, H., Långström, N. & Lichtenstein, P. Antipsychotics, mood stabilisers, and risk of violent crime. *The Lancet* **384**, 1206-1214 (2014).

40 Ekström, M. P., Bornefalk-Hermansson, A., Abernethy, A. P. & Currow, D. C. Safety of benzodiazepines and opioids in very severe respiratory disease: national prospective study. *BMJ* **348**, g445 (2014).

41 Latvala, A., Kuja‐Halkola, R., D'onofrio, B. M., Larsson, H. & Lichtenstein, P. Cognitive ability and risk for substance misuse in men: genetic and environmental correlations in a longitudinal nation‐wide family study. *Addiction* **111**, 1814-1822 (2016).

42 Sariaslan, A. *et al.* The impact of neighbourhood deprivation on adolescent violent criminality and substance misuse: A longitudinal, quasi-experimental study of the total Swedish population. *Int. J. Epidemiol.* **42**, 1057-1066, doi:10.1093/ije/dyt066 (2013).

43 D'Onofrio, B. M. *et al.* Familial confounding of the association between maternal smoking during pregnancy and offspring criminality: a population-based study in Sweden. *Arch. Gen. Psychiatry* **67**, 529-538 (2010).

44 Salvatore, J. E. *et al.* Alcohol use disorder and divorce: evidence for a genetic correlation in a population‐based Swedish sample. *Addiction* **112**, 586-593 (2017).
